# Artificial Intelligence in Image Assisted Radiation Oncology

**DOI:** 10.3390/cancers18111715

**Published:** 2026-05-25

**Authors:** He Wang, Yao Zhao, Xinru Chen, Brigid McDonald, Yunxiang Li, Jiacheng Xie, Dong Joo Rhee, Tze Yee Lim, Tucker J. Netherton, Jack Phan, Michael T. Spiotto, Mu-Han Lin

**Affiliations:** 1 Radiation Physics, University of Texas M.D. Anderson Cancer Center, Houston, TX 77030, USA; yzhao15@mdanderson.org (Y.Z.); xinru.chen@bcm.edu (X.C.); bmcdonald@mdanderson.org (B.M.); drhee1@mdanderson.org (D.J.R.); tlim@mdanderson.org (T.Y.L.); tnetherton@mdanderson.org (T.J.N.); 2 Radiation Oncology, University of Texas Southwestern Medical Center, Dallas, TX 75390, USA; yunxiang.li@utsouthwestern.edu (Y.L.); jiacheng.xie@utsouthwestern.edu (J.X.); mu-han.lin@utsouthwestern.edu (M.-H.L.); 3 Radiation Oncology, University of Texas M.D. Anderson Cancer Center, Houston, TX 77030, USA; jphan@mdanderson.org (J.P.); mtspiotto@mdanderson.org (M.T.S.)

**Keywords:** radiotherapy, artificial intelligence, automation, cancer detection, outcome analysis, radiomics, deep learning, machine learning, image assistance

## Abstract

Advanced imaging is the cornerstone of modern radiation oncology, contributing to each phase of patient care, from diagnosis and treatment planning to delivery and follow-up. It has evolved from providing purely geometric guidance to enabling biological and dynamic precision, capturing detailed spatial and functional information about tumors and surrounding tissues. This progress has also generated vast amounts of complex data that remain largely underexplored. AI-based methods have shown promises to unlock the potential of these data, ensuring quality and standardization while extracting previously inaccessible insights. AI-driven tools can enhance accuracy, efficiency, and personalization of radiation oncology through precision diagnosis, automated segmentation, adaptive treatment planning, real-time image guidance, and predictive response assessment. In this review, we conducted a systematic bibliometric analysis of relevant literature published in the last decade and explored current advancements in AI and radiomics applications across radiation oncology. We also addressed ongoing challenges, such as data heterogeneity, model interpretability, and clinical implementation, and discussed future directions for integrating AI-powered imaging solutions into routine practice to advance precision cancer care.

## Simple Summary

Advanced imaging is essential to modern radiation oncology, integral to every phase of patient care. This generates vast, complex data that remain underexplored. The integration of artificial intelligence (AI) has shown promise to unlock this potential, ensuring quality and standardization while extracting previously inaccessible insights. This review surveys current advancements in AI and radiomics across radiation oncology, addresses ongoing challenges, and discusses future directions for embedding AI-powered imaging solutions into routine practice to advance precision cancer care.

## 1. Introduction

From the discovery of X-ray in 1895 to today’s advanced megavoltage techniques, radiotherapy has made profound contributions to cancer treatment over the past 130 years. The fundamental therapeutic goal is to deliver a tumoricidal dose to the target volume while minimizing radiation exposure to surrounding healthy tissue and critical organs at risk (OARs). Because radiation effects are inherently spatially dependent and cumulative, radiotherapy is highly sensitive to geometric and dosimetric uncertainties, which can directly influence tumor control probability and normal tissue complication probability. Accuracy and precision therefore lie at the heart of radiotherapy, particularly for modern highly conformal techniques such as intensity-modulated radiotherapy (IMRT), stereotactic body radiotherapy (SBRT) and proton therapy, which rely on tighter margins and steeper dose gradients.

**Figure 1:**
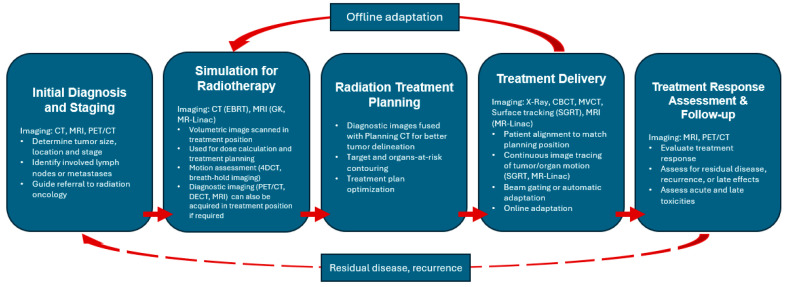
Workflow of radiotherapy and contributions of imaging. EBRT—external beam radiotherapy; GK—Gamma Knife; DECT—dual-energy CT; SGRT—surface guided radiotherapy; CBCT—cone beam CT; MVCT—megavoltage CT.

Technical advancements over recent decades have markedly enhanced the precision and accuracy of radiation therapy. These achievements would not have been possible without parallel advances in medical imaging [1]. A typical radiotherapy workflow is shown in Figure 1, highlighting the essential role of imaging in radiotherapy treatment and planning. As illustrated, imaging has become deeply integrated into every phase of radiotherapy through multiple modalities.

Large-scale medical imaging resources are already available in contemporary clinical practice. Using head-and-neck cancer (HNC) as an example, GLOBOCAN 2022 reported approximately 940,000 new HNC cases and 480,000 related deaths worldwide [2]. Oral, pharyngeal, and laryngeal squamous cell carcinomas (SCCs) account for nearly 90% of these cases, and almost 75% of patients with head-and-neck SCC (HNSCC) will benefit from radiotherapy as part of their treatments [3]. A contemporary HNC radiotherapy patient may generate multiple imaging datasets throughout the course of care, including diagnostic CT, MRI, and PET scans for staging and target definition; a simulation CT for treatment planning; daily or weekly cone-beam CT (CBCT) for setup verification; additional CT or MRI scans for adaptive replanning; and post-treatment diagnostic imaging for response assessment and follow-up at multiple time points after completion of radiotherapy. Collectively, these imaging datasets contain substantially more information than is currently leveraged in routine clinical practice and represent a rich resource for advanced data mining, quantitative imaging, and clinical decision support.

Artificial intelligence (AI), particularly machine learning (ML) and deep learning (DL), offers a powerful tool to utilize this wealth of data to develop predictive models which can expedite decision-making, treatment planning and response assessment [4,5]. It involves learning complex rules and patterns from historical data, which are then used to predict outcomes or automate and simplify complex tasks. AI enables radiomics [6,7] and outcome modeling [8,9] by extracting high-dimensional quantitative imaging features associated with tumor control, normal tissue toxicity, and treatment response. Moreover, AI excels in automation of clinical tasks such as target and OAR detection and delineation, treatment planning and treatment delivery. When integrated across the workflow, AI enables standardized and efficient processes while supporting high-precision and personalized radiotherapy tailored to an individual patient’s anatomy and disease characteristics.

In recent years, research on the application of AI in radiation oncology has expanded rapidly, alongside the development of commercial AI-enabled tools. Examples include Varian Ethos (Varian Medical Systems, Palo Alto, CA, USA) [10] and TheraPanacea ART-Plan (TheraPanacea, Paris, France) [11] for adaptive radiotherapy (ART), RayStation Treatment Planning System (RaySearch Laboratories, Stockholm, Sweden) [12] for auto-segmentation and intelligent treatment planning, MVision AI (MVision, Helsinki, Finland) [13] and Limbus AI (Limbus AI Inc., Regina, SK, Canada) [14] for automated contouring, and Imbio Lung Suite (Imbio LLC, Minneapolis, MN, USA) [15] and RadiomiX (OncoRadiomics SA, Liège, Belgium) [16] as radiomics platforms for extracting advanced imaging biomarkers in oncology. The potential of AI continues to be actively explored to improve efficiency and consistency in clinical workflows.

A particularly important recent development is the emergence of generative AI, including generative adversarial networks, diffusion models, and related foundation-model approaches. These advances have broadened biomedical imaging research from image analysis toward image synthesis, reconstruction, modality translation, data augmentation, and workflow support. In this context, Hsieh et al. emphasized that the growing use of generative AI in biomedical imaging and scientific authorship raises critical questions regarding transparency, disclosure of AI assistance, accountability for generated content, reproducibility of AI-assisted workflows, and research governance. These considerations are essential to the responsible development and reporting of AI-enabled image-assisted radiation oncology research [17].

Despite these advances, significant limitations and concerns remain. These include the “black-box” nature of many AI models, data heterogeneity across institutions, algorithmic bias, and potential patient safety risks. As a result, only a limited number of AI tools have been fully integrated into routine clinical practice, particularly for high-stakes clinical decision-making where transparency, robustness, and interpretability are essential. Nevertheless, clinical adoption is steadily increasing as recommendations for good practices, governance, and lifecycle management continue to emerge in the software as a medical device space [18].

In this review, we comprehensively examine AI applications in radiation oncology, with a focus on both clinical practice and related research aimed at improving radiation treatment and outcomes. We performed a PubMed search and bibliometric analysis to evaluate publication trends, keyword evolution, and author contributions. We then summarized current AI and radiomics applications across imaging-dependent radiotherapy workflow, from cancer detection and radiation treatment planning to quality assurance, treatment delivery, and outcome analysis. The associated challenges and future directions were also discussed.

## 2. AI Publications in Radiation Oncology Field

We performed a systematic search of PubMed combining four radiotherapy synonyms (‘radiation therapy’, ‘radiotherapy’, ‘radiation treatment’, ‘radiation oncology’) with four AI synonyms (‘artificial intelligence’, ‘AI’, ‘machine learning’, ‘deep learning’) in Title/Abstract fields, yielding 16 Boolean queries of the form (RT term[Title/Abstract]) AND (AI term[Title/Abstract]). The search was performed via the NCBI E-utilities API, restricted to publication dates between 2015 and 2025, and finalized after the close of the 2025 calendar year. Records were retrieved across all 16 queries and deduplicated by PMID, yielding 2064 unique publications that constitute the bibliometric corpus analyzed in this section. The 2025 publication count of 451 reflects a complete calendar year. Bibliometric processing—keyword frequency analysis, year-to-year Jaccard similarity, PELT change-point detection, author productivity and collaboration network analysis, and community detection—was performed in Python v3.10 (NetworkX, ruptures, SciPy). This corpus is distinct from the narrative review corpus cited in Section 3, Section 4, Section 5, Section 6, Section 7, Section 8, Section 9 and Section 10, which were selected through expert-driven targeted searches and citation tracking and may include records outside PubMed.

Author keywords from PubMed records were lowercased and filtered against a stop-list of generic demographic descriptors (humans, male, female, adult, aged, middle aged, young adult, adolescent, child, infant, animals). No further normalization (stemming, synonym merging, or controlled-vocabulary mapping) was applied; we acknowledge as a limitation that this leaves variant forms (e.g., ‘CNN’/‘convolutional neural network’, ‘AI’/‘artificial intelligence’, ‘auto-segmentation’/‘automatic segmentation’) as separate keywords in the analysis.

### 2.1. Publication Trend Analysis

**Figure 2:**
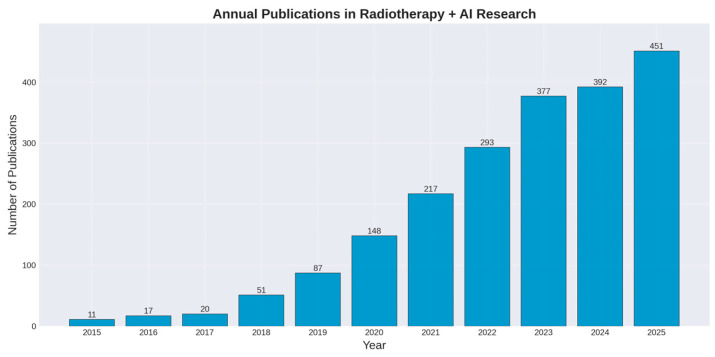
Annual publication trends of AI in radiation oncology from 2015 to 2025.

Figure 2 illustrates the annual publication volume trends of AI in radiation oncology from 2015 to 2025, demonstrating a rapid growth trend in this field. In 2015, only 11 related papers were published, while by 2025, the annual publication volume had reached 451 papers, representing an approximately 41-fold increase. Growth accelerated markedly after 2019, with publications increasing from 87 papers in 2019 to 217 papers in 2021, corresponding to an annual growth rate exceeding 50% and reflecting the maturation and broader adoption of deep learning in radiotherapy.

### 2.2. Journal Distribution Analysis

**Figure 3:**
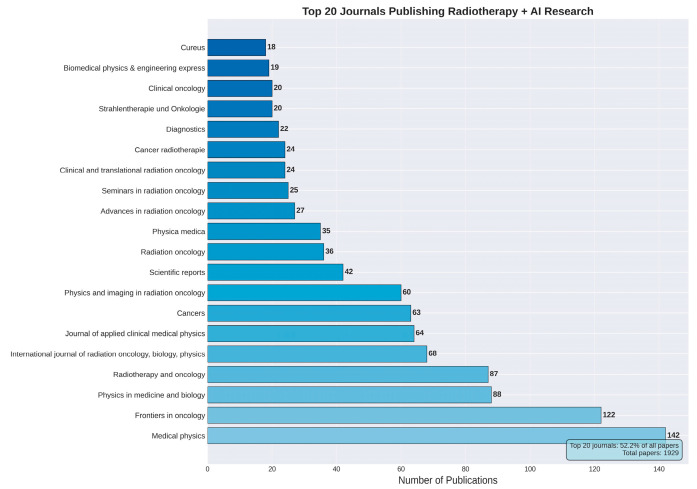
Distribution of top 20 journals publishing AI in radiation oncology research.

The distribution of journals publishing AI research in radiation oncology reflects both the academic influence and research direction of this field. As shown in Figure 3, a total of 440 journals have published relevant research, with the top 20 journals accounting for 52.2% of all papers, indicating a certain degree of concentration. *Medical Physics* ranks first with 142 papers, followed by *Frontiers in Oncology* (122 papers) and *Physics in Medicine and Biology* (88 papers). Notably, the top 20 journals span multiple disciplines including medical physics, radiation oncology, medical imaging, and artificial intelligence, highlighting the interdisciplinary nature of this research field.

### 2.3. Keyword Analysis

#### 2.3.1. Overall Keyword Distribution

**Figure 4:**
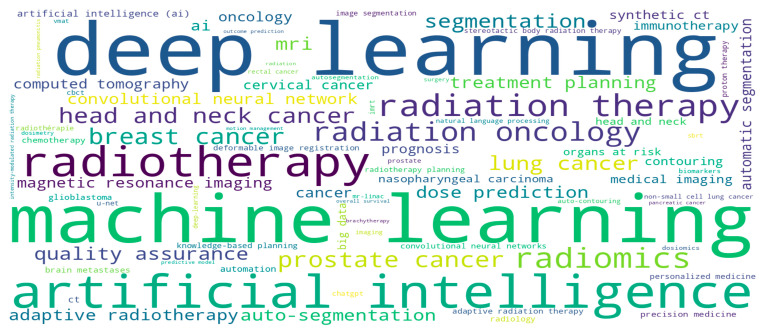
Keyword cloud of AI in radiation oncology research.

Through analysis of keywords from all papers, we constructed a keyword cloud (Figure 4). The figure clearly shows that “deep learning,” “machine learning,” “radiotherapy,” and “artificial intelligence” are the most frequently occurring keywords, reflecting the core research content of this field. Other high-frequency keywords include “segmentation,” “radiation oncology,” “treatment planning,” “radiomics,” and “dose prediction,” which encompass the main application directions of AI in radiotherapy.

#### 2.3.2. Temporal Evolution of Keywords

**Figure 5:**
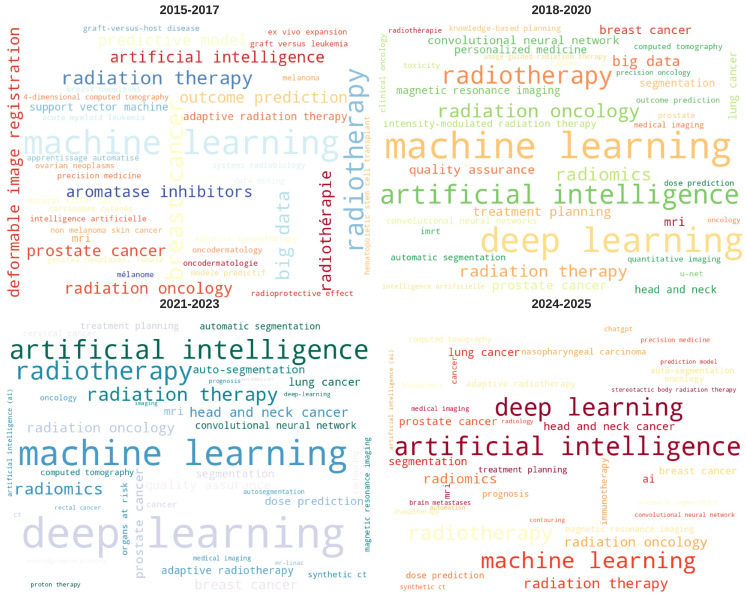
Keyword evolution in AI radiation oncology research across different periods.

To understand the evolution of research hotspots, we divided the study period into four phases for keyword evolution analysis (Figure 5). These phase boundaries are justified by two complementary data-driven analyses. First, Pruned Exact Linear Time (PELT) change-point detection on log-transformed annual publication counts identifies a single statistically robust change-point at the end of 2017, stable across penalty values from 0.5 to 3.0, which directly supports the 2017/2018 boundary. Second, year-to-year Jaccard similarity of the top 30 author keywords reveals the largest vocabulary shift at 2017 → 2018 (1 − Jaccard = 0.91), independently confirming this primary breakpoint. The remaining year-to-year transitions show a clear two-stage pattern: vocabulary turnover is high during 2018–2020 (consistent with rapid methodological churn) and declines during 2021–2025 (consistent with consolidation around established techniques). Each phase is further supported by a combination of growth-rate, keyword-distribution, and external-milestone evidence, as summarized below.

**Phase 1 (2015–2017):** Pre-DL/Traditional ML era (n = 48 publications). Machine learning is the most frequent keyword (n = 11), and traditional-ML methods dominate; support vector machine appears in the top 15. Distinctive keywords relative to later phases include predictive model, outcome prediction, deformable image registration, adaptive radiation therapy, big data, and breast cancer (the dominant clinical site of this early period). The phase reflects an exploratory era in which traditional ML was applied to specific radiotherapy subproblems.

**Phase 2 (2018–2020):** DL Take-off (n = 286). The +155% YoY publication growth in 2018 marks the decisive transition supported by PELT analysis, coinciding with the widespread maturation of GPU computing, ImageNet-pretrained backbones, U-Net-style segmentation architectures, and the large public segmentation challenges run through MICCAI. For the first time, deep learning (n = 62) and artificial intelligence (n = 52) enter the top 3 keywords. Distinctive emerging terms include intensity-modulated radiation therapy (lift 10.9× relative to other phases), personalized medicine (4.5×), head-and-neck (4.0×), treatment planning (3.0×), convolutional neural network (2.9×), quality assurance (2.2×), big data (2.1×, retained from Phase 1 framing), and magnetic resonance imaging (2.0×).

**Phase 3 (2021–2023):** Clinical Translation (n = 887). Annual growth rate transitions from the +71% explosive regime of Phase 2 to a +47%/+35%/+29% consolidation regime, reflecting a shift from architecture-development papers toward clinical-application papers. Deep learning itself becomes a distinctive keyword for this phase (lift 2.1×, n = 277), confirming this as the era when DL became the methodological foundation of the field. Other distinctive keywords are dominated by clinical sites and clinical workflows: head-and-neck cancer (lift 3.0×), auto-segmentation (2.8×), dose prediction (2.4×), and quality assurance (2.1×). Externally, this period coincides with the broad adoption of transformer architectures (ViT 2020[19], Swin Transformer 2021 [20]) and the transition of AI radiotherapy methods from pure methodology research toward clinical translation.

**Phase 4 (2024–2025):** Foundation Models/Maturation (n = 843). For the first time in the corpus, the generic term artificial intelligence (n = 227) overtakes deep learning (n = 182) as the most frequent author keyword, reflecting a broader framing shift in the medical-AI literature that coincides with the rise in foundation models and large language models in adjacent fields (ChatGPT, late 2022; GPT-4, March 2023). The abbreviation AI itself also emerges as a distinctive Phase-4 keyword (lift 4.3×, n = 25). Annual growth rate moderated relative to Phase 3 (2024 = +4% YoY, +15% in 2025), consistent with field maturation; nonetheless, the per-year publication rate in Phase 4 (≈420 papers/year) is approximately 40% higher than the Phase 3 average (≈295 papers/year), reflecting the continuing expansion of the literature. Other new distinctive keywords reflect the integration of imaging AI with treatment-outcome research and an expansion to broader oncology framing: immunotherapy (lift 5.0×), oncology (2.7×), segmentation (2.1×, broader and distinct from the more methodology-specific auto-segmentation of Phase 3), and lung cancer (2.1×).

Across the four phases, the boundaries are thus supported by complementary forms of evidence: statistical change-point detection, year-to-year keyword vocabulary turnover, growth-rate transitions, phase-distinctive keyword shifts, and identifiable technical/clinical milestones. Together, these speak to a genuine evolution of the field’s research focus, not merely to publication-volume growth.

### 2.4. Author Collaboration Network Analysis

#### 2.4.1. Distribution of Highly Productive Authors

**Figure 6:**
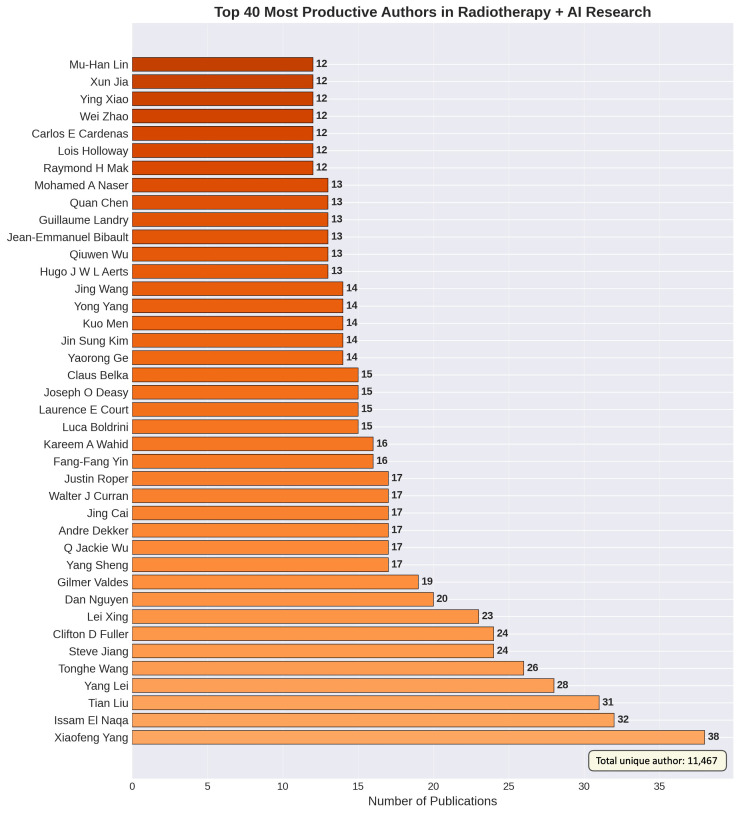
Top 40 most productive authors in AI radiation oncology research as determined by the keyword search.

Figure 6 shows the 40 most prolific authors in this field, as determined by the keyword search. Xiaofeng Yang ranks first with 38 papers, followed by Issam El Naqa (32 papers) and Tian Liu (31 papers). To place this concentration in context, we compared the observed productivity distribution against Lotka’s law, the standard scientometric reference for author productivity. The fitted exponent is α = 2.69 (R^2^ = 0.975), steeper than Lotka’s classical α = 2.0, indicating that a small number of highly prolific research groups drive a disproportionate share of the literature while the long tail of single-publication authors is broader than the classical prediction. Authors were identified by the concatenation of first and last names recorded in PubMed, without explicit ORCID-based disambiguation; this may underestimate productivity for authors with name variants and should be noted as a methodological limitation.

#### 2.4.2. Author Collaboration Network

**Figure 7:**
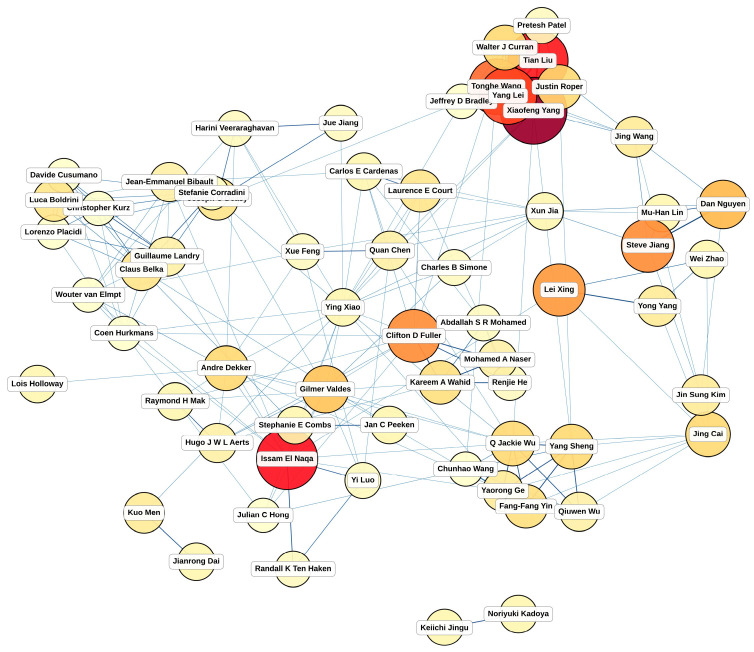
Collaboration network of highly productive authors.

The author collaboration network diagram (Figure 7) illustrates the collaborative relationships among the 70 authors with 10 or more publications (after removing two isolates: 68 nodes, 225 edges). Collaboration ties were defined as co-authorship on the same publication, with edge weights equal to the number of jointly authored papers. The visualization is restricted to the giant component and uses a force-directed layout; node size represents publication volume and edge thickness reflects collaboration strength.

Quantitative network metrics characterize the field’s collaborative structure. Network density is 0.099 (sparse; mean degree 6.6). The average clustering coefficient is 0.593, approximately 6.0× the random-graph baseline, indicating strong community organization. Global transitivity is 0.500, and modularity Q = 0.745, exceeding the conventional Q ≥ 0.7 threshold for very strong community structure.

Three centrality measures (degree, betweenness, eigenvector) surface complementary roles among highly productive authors. Eigenvector centrality is highest for Xiaofeng Yang (0.465), Tian Liu (0.446), and Yang Lei (0.429), the densely interconnected core of one of the largest communities. Betweenness centrality highlights bridge authors who connect otherwise separate communities—these broker roles are critical for cross-pollination of methods across clinical application areas.

Inspection of the 10 detected communities shows that they map cleanly onto recognized PI-led research groups. The eight largest communities are anchored by: an outcome-modeling cluster centered on Issam El Naqa, Andre Dekker, and Gilmer Valdes (n = 14); the LMU Munich MR-guided RT group around Claus Belka, Luca Boldrini, and Stefanie Corradini (n = 14); the MD Anderson head-and-neck cluster around Clifton Fuller, Kareem Wahid, and Laurence Court (n = 9); the Emory deep-learning group around Xiaofeng Yang, Tian Liu, and Yang Lei (n = 8); a Duke Knowledge-Based Planning cluster around Yang Sheng, Q. Jackie Wu, and Fang-Fang Yin (n = 7); the UT Southwestern deep-learning cluster around Steve Jiang, Dan Nguyen, and Jing Wang (n = 6); a Stanford methodology cluster around Lei Xing and Wei Zhao (n = 3); and a Dana-Farber/MGH radiomics cluster around Hugo J. W. L. Aerts, Raymond H. Mak, and Danielle S. Bitterman (n = 3).

Together, these metrics characterize the field as highly clustered and strongly modular, with a small set of broker authors connecting otherwise locally tight research communities. The high modularity (Q = 0.745) raises a practical concern: while internal community cohesion supports rapid methodological iteration within groups, the relatively low cross-community density may slow the diffusion of new methods across institutions, potentially contributing to the well-documented difficulty of reproducing imaging-AI results across data domains.

### 2.5. Research Hotspots

Based on the above bibliometric and keyword-evolution analysis, several major research hotspots in AI for radiation oncology can be identified. Each is anchored in the quantitative keyword analysis of Section 2.3, with keyword-frequency trajectory and remaining limitations noted:

- **Automatic Segmentation.** Among the most widely applied areas, with combined keyword frequencies for ‘segmentation’, ‘auto-segmentation’, and ‘organ at risk’ growing from rank 14 in Phase 1 to rank 7 in Phase 3 (n = 80 in Phase 3 alone). Deep learning models have achieved substantial progress in automated delineation of OARs and tumors. Clinical translation, however, remains uneven: cross-institutional generalization, contouring variability when target boundaries are ambiguous (e.g., GTV in head-and-neck), the absence of harmonized regulatory pathways for adaptive segmentation tools, and limited prospective dosimetric validation continue to limit routine adoption.
- **Radiomics Analysis.** ‘Radiomics’ first appears as a top 15 keyword in Phase 2 and rises to rank 4 in Phase 3 (n = 112). AI-driven extraction of quantitative imaging features supports prognosis prediction and treatment decision-making. However, reproducibility remains a major barrier: sensitivity to acquisition parameters, lack of standardized feature-extraction pipelines, small single-institution cohorts, and limited independent external validation restrict clinical translation.
- **Dose Prediction and Optimization.** ‘Dose prediction’ emerges as a distinctive keyword in Phase 3, rising to rank 6 (n = 68), reflecting the clinical-translation shift in this period. AI models for predicting dose distribution and optimizing treatment plans have shown promise in reducing planning time, but challenges persist in ensuring physical deliverability of predicted plans, handling complex multi-criteria optimization trade-offs, and validating across treatment techniques and disease sites.
- **Image Reconstruction and Enhancement**. Keywords related to image reconstruction, including ‘computed tomography’ and ‘image reconstruction’, are prominent in Phase 2 and persist through Phase 3. Deep learning for low-dose CT reconstruction and sparse-view reconstruction is increasingly prevalent. Key limitations include the risk of hallucinated anatomical structures, dependence on training-distribution coverage, loss of interpretability relative to physics-based algorithms, and limited evaluation under clinically realistic distribution-shift conditions.
- **Treatment Response Prediction**. ‘Outcome prediction’ appears among distinctive keywords in Phase 1 and re-emerges in Phase 3 with higher frequency, reflecting a sustained interest that has matured from traditional ML to DL approaches. Integration of multimodal data to predict radiotherapy responses supports personalized treatment decisions, but existing models are largely retrospective, trained on heterogeneous endpoint definitions, and rarely validated in prospective clinical workflows.

### 2.6. Summary of Publication Search

The bibliometric analysis highlights the rapid development of AI in radiation oncology. From 2015 to 2025, this field has experienced a transformation from an exploratory phase to a period of rapid growth, with research hotspots gradually shifting from basic algorithm development toward clinical applications. The diversity of journal distribution and the complexity of author collaboration networks reflect the interdisciplinary nature and international collaboration trends within the field. As technologies mature and clinical demand increases, AI in radiation oncology is expected to maintain strong growth, particularly in areas such as personalized medicine and precision radiotherapy.

## 3. AI in Imaging of Radiation Oncology

### 3.1. Cancer Detection in Diagnostic Phase

Although cancer detection and screening occur upstream of radiation oncology decision-making, they critically influence which patients are referred for radiotherapy and at what stage of disease. These factors, in turn, shape the technical and biological demands of subsequent treatment planning. The examples in this section illustrate how AI-driven diagnostic imaging affects the patient population entering radiotherapy workflows, with downstream implications for treatment volume, fractionation strategies, and outcome prediction. Studies of diagnostic AI without direct relevance to radiotherapy are beyond the scope of this review.

#### 3.1.1. Early Cancer Detection and Screening Models

Early detection is essential for improving cancer outcomes but remains challenging, as early-stage lesions are often small and inconspicuous. Effective screening must balance high sensitivity with low false-positive rates. Gillies and Schabath [21] highlighted radiomics as a strategy to extract quantitative features that reveal subtle patterns not readily apparent on visual assessment. Thanoon et al. [22] reviewed the DL techniques for lung cancer screening and diagnosis based on CT images, demonstrating the capability of three-dimensional image analysis for automated nodule identification and malignancy risk stratification. This advancement from abnormality detection to risk assessment has important implications for optimizing subsequent diagnostic and therapeutic decisions. However, the authors also raised concerns regarding the use of heterogenous datasets for model training, emphasizing that careful preprocessing—including image standardization, annotation consistency and cohort balancing—is essential to achieve precise and effective lung cancer screening.

AI has also been investigated in breast cancer screening. In a large multicenter study, McKinney et al. [23] evaluated an AI system across diverse populations and healthcare settings, demonstrating reductions in false-positive rates of 5.7% (US dataset) and 1.2% (UK dataset), and in false-negative rates of 9.4% (US) and 2.7% (UK), in independent retrospective evaluations. These findings highlight the potential to decrease unnecessary follow-ups and patient anxiety while improving early detection, though the cross-cohort variation underscores the well-known generalization challenge of screening AI, and these gains have not yet been confirmed in prospective trials. For pancreatic cancer, where prognosis remains poor, early detection is particularly important. Placido et al. [24] developed a temporal prediction model using disease trajectory data that identified high-risk patients up to three years before clinical diagnosis. The model was trained on Danish national-registry data and showed reduced performance when transferred to US Veterans Affairs data, illustrating both the promise of trajectory-based AI for early detection and the substantial domain-shift challenge of cross-health-system deployment. This work illustrates how AI can help uncover hidden patterns of disease development from large-scale clinical data.

#### 3.1.2. Radiomics for Feature Extraction

Radiomics, as an emerging medical image analysis technique, can extract large amounts of quantitative features from standard scans like CT, MRI, and PET. It detects subtle patterns invisible to human eyes, acting as imaging biomarkers to reveal underlying tumor biology and microenvironment.

In recent years, the rapid development of AI technology, particularly DL, has significantly advanced radiomics in radiation oncology. Traditional radiomics relies on hand-crafted features that are mathematically defined: shape descriptors (e.g., compactness, sphericity), first-order intensity statistics, and second-order texture features computed from gray-level co-occurrence and run-length matrices. These features are reproducible in principle, but their values are sensitive to image acquisition parameters and preprocessing choices (resampling, intensity quantization, ROI delineation), which has motivated harmonization efforts such as the Image Biomarker Standardization Initiative (IBSI) [25]. Deep learning, by contrast, learns task-specific representations from the images themselves, enabling discovery of patterns that hand-crafted descriptors may miss, at the cost of reduced interpretability and much higher data requirements.

A review by Kocher et al. summarized DL-enhanced radiomics studies for brain tumor diagnosis with reported accuracies generally in the 80–90% range across heterogeneous tasks (e.g., glioma grading, IDH mutation prediction, MGMT methylation status), datasets (single-center vs. multi-center, sample sizes ranging from tens to hundreds of patients), and validation strategies (k-fold cross-validation vs. external test set). Direct comparison across these reported accuracies is not meaningful, and the figures should be interpreted as a proxy for active research progress rather than head-to-head performance [26]. Similarly, Dreher et al. reviewed the radiomics application in the diagnosis and treatment of liver tumors [27], and indicated that radiomics can support more precise individualized treatment planning and decision-making in SBRT. Together, these studies demonstrated the broad clinical utility of radiomics.

Beyond diagnosis and treatment planning, radiomics is particularly valuable for linking imaging features with underlying tumor biology. Studies have reported associations between radiomics features and tumor gene expression profiles [28,29,30] under the concept of “radiogenomics”, highlighting the potential for non-invasive tumor molecular typing. Radiomic features have also demonstrated prognostic value to predict survival outcomes with performance comparable to and complementary to traditional clinical factors [31,32]; the most robust signals to date come from combined radiomics–clinical nomograms rather than radiomics alone. Radiogenomic and prognostic radiomics studies do, however, face well-documented reproducibility challenges—small single-institution cohorts, the lack of standardized feature-extraction pipelines, sensitivity to scanner and acquisition variability, and limited independent validation—which currently limit clinical translation [31]. These advances position radiomics as a promising research tool for precision oncology, but they underscore the need for harmonization, prospective validation, and transparent reporting. The emergence of immune checkpoint inhibitors has improved outcomes for several cancers, but accurately predicting treatment response remains challenging. Conventional biomarkers such as PD-L1 expression require tissue biopsy and have limited predictive performance. In this context, AI-based radiomics demonstrate unique advantages. Trebeschi et al. developed a DL model based on CT images that can predict non-small-cell lung cancer (NSCLC) response to immunotherapy with 83% accuracy [33]. The distinctive feature of this work is that the model incorporates imaging features from both the tumor and surrounding tissue, thereby capturing aspects of the peritumoral immune microenvironment. Furthermore, Mu et al. adopted a multimodal imaging strategy [34]. They combined PET/CT radiomics features with clinical factors to establish a predictive model of both treatment response and risk of severe immune-related adverse events. This dual predictive capability highlights the potential of radiomics to optimize patient selection and safety monitoring in personalized immunotherapy. These radiomics studies bridge the gap between feature extraction and clinical application. As datasets expand and methodologies mature, radiomics is expected to play an increasing role in immunotherapy.

### 3.2. Image Acquisition

Medical image reconstruction is a fundamental component of radiation therapy, as high-quality imaging is crucial for accurate target delineation and dose calculation. With the development of AI technology, the field of image reconstruction is experiencing an important transition from traditional iterative algorithms to DL methods. This transition not only improves image quality but more importantly changes our understanding and approach to solving image reconstruction problems.

#### 3.2.1. Sparse-View Image Reconstruction

The development of sparse-view CT reconstruction addresses the critical clinical need to maintain image quality while reducing radiation dose. Traditional filtered back projection (FBP) algorithms produce severe streak artifacts under sparse sampling conditions—a physical limitation long considered insurmountable. The emergence of DL methods has substantially mitigated this limitation; although, new concerns have arisen, including hallucination of plausible but non-existent anatomical structures, loss of interpretability of the reconstruction process, and dependence on training-distribution coverage. Distribution shifts at inference time (e.g., scanner change, anatomy outside the training distribution) can degrade reconstruction quality in ways that are not always evident from the output image alone [35]. Furthermore, sparse-view reconstruction can also aid resource-constrained regions where traditional CT scanners are unavailable. Sun et al. demonstrated that CT volumes could be reconstructed from just a few planar X-rays, expanding access to radiotherapy [36].

The DD-Net method proposed by Zhang et al. reframed sparse-view reconstruction as an image-domain restoration task applied to an initial FBP reconstruction [37]. This “reconstruct first, then restore” strategy combines physical reconstruction with a data-driven prior. More broadly, modern DL-based CT reconstruction approaches retain elements of both formulations: pure image-domain restoration (post-processing of FBP), pure projection-domain learning, and hybrid model-based or unrolled approaches that explicitly enforce the imaging physics. The conceptual contribution of this line of work is therefore not to replace inverse-problem formulations with restoration, but to allow data-driven priors to complement physics-based forward models. However, it does not fully utilize the physical constraints of the acquired data. To address this, Liu et al. proposed a geometry-aware attenuation learning method [38]. Their network models projection geometry relationships and can reconstruct images close to full-sampling quality using only 1/4 of the projection data. This advancement demonstrates that embedding domain knowledge into network architecture is a powerful strategy for improving performance.

The CT-SDM model developed by Yang et al. [39] represents a new generation of reconstruction methods. Unlike traditional deterministic reconstruction, this model utilizes diffusion probabilistic models to generate multiple reconstructions, offering the ability to quantify reconstruction uncertainty. More importantly, its generalization ability shows the potential to adapt to different sampling rates without retraining. Additionally, the results of DL-Sparse-View CT Grand Challenge sponsored by the American Association of Physicists in Medicine (AAPM) highlighted that the winning methods employ hybrid strategies, combining data-driven learning with physical models, proving that domain knowledge remains essential in the AI era [40].

The field continues to advance through comparative evaluations of different architectural approaches. Bottini et al. [41] compared generative adversarial networks (GANs) and CNN-based implicit neural representations (CNN-INRs) for generating synthetic spine CT images from biplanar radiographs. Their work provides valuable insights into the strengths and limitations of different DL architectures for sparse-view reconstruction, informing method selection for specific clinical applications. Similarly, Lai et al. [42] used self-supervised neural fields to reconstruct knee CT volumes from biplanar X-rays, demonstrating that self-supervised learning can effectively leverage the inherent image structure without large labeled datasets—an advantage in data-limited clinical settings.

#### 3.2.2. Four-Dimensional (4D) Image Acquisition

4D imaging arose from the recognition that organs move continuously, whereas conventional 3D imaging captures only static snapshots. For thoracic and abdominal tumors, respiratory motion can cause displacements of several centimeters, challenging precision radiotherapy. By adding a temporal dimension, 4D imaging enables motion-resolved assessment, though accurate modeling of respiratory motion remains a key challenge.

Thummerer et al. [43] addressed this by generating high-quality 4D synthetic CT from sparse-view CBCT for adaptive proton therapy, where density accuracy is critical. Using deep CNNs to learn the mapping relationship between sparse CBCT and high-quality CT, they achieved mean absolute error within 50 Hounsfield units (HUs) and 3%/3 mm gamma pass rates above 90% on a held-out test set, computed against high-quality 4D-CT reference plans. For clinical context, routine acceptance thresholds for proton dose calculation typically require at least 95% pass rate at 3%/3 mm; the reported performance therefore demonstrates feasibility but suggests further refinement is needed before unsupervised clinical deployment, particularly for high-dose-gradient regions and motion-management-critical anatomy.

To address the limited spatiotemporal modeling capacity of conventional CNNs, which rely on local convolutional kernels and have difficulty capturing long-range dependencies across the temporal dimension of the 4D volume, Cao et al. [44] introduced a Mask-Based Swin Transformer network (MBST). Window-based attention enables the model to relate distant respiratory phases without inflating receptive fields through deeper convolutional stacks, which is particularly relevant for reconstructing motion boundaries where information at one phase is informative for another. In lung cancer patients, this method showed superior performance in reconstructing motion boundaries, essential for accurate target delineation. Open challenges remain in enforcing temporal consistency across phases (avoiding flickering artifacts) and in disentangling intra-fraction motion from underlying anatomical change.

MRI-based 4D imaging faces distinct challenges as longer acquisition times make single breath-hold scans impractical. Murray et al. addressed this with MovieNet [45], which breaks traditional k-space consistency constraints and instead learns spatiotemporal correlations through deep networks. This approach is motivated by the observation that strict data consistency under highly undersampled conditions can introduce artifacts. By jointly leveraging spatial, temporal, and coil information, MovieNet significantly accelerated reconstruction, enabling free-breathing 4D MRI and improving patient comfort.

#### 3.2.3. Artifact Removal Techniques

Imaging artifacts significantly affect diagnostic and treatment planning accuracy. As they arise from different physical mechanisms, targeted correction is required. The introduction of DL technology not only improves artifact reduction but more importantly provides a unified framework for handling various types of artifacts.

**Metal Artifact Reduction**. Metal-induced streak artifacts in CT remain a persistent clinical challenge, degrading image quality and compromising dose-calculation accuracy. Koike et al. [46] applied a CycleGAN-based unpaired learning approach that learned mappings between the two domains of with and without artifact, without requiring perfectly paired datasets. The application to head-and-neck radiotherapy showed improved image quality and dose-calculation accuracy. Zhang and Yu [47] proposed a CNN-based metal artifact reduction (CNN-MAR) method that fused the information from the original and corrected images to suppress artifacts, which showed promising performance in complex, multi-implant scenarios. Liao et al. [48] introduced an artifact disentanglement network (ADN), treating metal artifacts as independent components mixed with tissue signals. By learning to disentangle these components, ADN removed streak artifacts while preserving soft tissue detail, demonstrating clinical value in patients with hip prostheses.

**Motion Artifact Correction**. Motion artifacts are complex and heterogeneous, ranging from rigid to nonrigid deformation, and from periodic respiration to random patient movement. Küstner et al. [49] proposed two DL-based networks for retrospective correction of motion-affected MRI without requiring external motion tracking or specialized acquisition protocols, demonstrating sufficient quality improvement across various types of motion-induced artifacts. Additional studies showed that GAN-based approaches offer powerful data-driven solutions, particularly for complex nonrigid motion scenarios [50,51]. A comprehensive review by Spieker et al. summarized deep learning-based strategies [52] for MRI motion correction, including data usage, architectures, training and evaluation methods, as well as challenges and limitations. Careful validation remains essential prior to clinical adoption.

### 3.3. Image Synthesis

Image synthesis represents a meaningful methodological shift in radiation therapy workflow—from passively accepting limitations of available imaging to actively generating clinically relevant information. Concrete clinical-adoption examples include MR-only prostate radiotherapy on the Elekta Unity and ViewRay/MRIdian MR-Linac systems, and CBCT-based synthetic-CT workflows on the Varian Ethos system, both of which have entered routine clinical use at multiple institutions. From MRI-only workflows to on-line adaptive therapy, image synthesis is increasingly reshaping the boundaries of modern radiotherapy practice.

#### 3.3.1. CT Synthesis from MRI

Over the past decade, MRI-guided radiotherapy has emerged as a clinically established approach to improve treatment precision and outcomes for selected anatomical sites. The drive toward MRI-only radiotherapy has accelerated the development of synthetic CT (sCT), aiming to reduce radiation exposure, simplify workflow, and eliminate registration errors. Meanwhile, for CBCT-based ART, the sCT has also shown to be a powerful tool in the evaluation of treatment quality. The core challenge is to generate accurate electron density information from MRI or CBCT for reliable dose calculation.

Bahloul et al. [53] reviewed advanced sCT generation approaches and highlighted the potential of DL architectures for more accurate and patient specific sCTs. The SynthRAD2023 Grand Challenge marked a milestone in this field [54]. According to the official challenge leaderboard, top-performing methods achieved approximately 16–28 HU mean absolute error (MAE) for pelvis and brain regions, respectively. For clinical context, dose calculation on synthetic CT is typically considered acceptable when the resulting dose distribution agrees with reference CT-based dose to within approximately 1% in target dose metrics and within clinical OAR tolerances, which corresponds approximately to soft-tissue MAE thresholds of 30–50 HU and tighter requirements in bone. The reported pelvis MAE therefore approaches clinically acceptable performance, while accuracy in regions with metal implants, air-tissue interfaces, and outside-FOV (field-of-view) structures remains a known challenge More importantly, the competition promoted method standardization and benchmark establishment, accelerating clinical translation.

Single MRI sequences often lack sufficient information for accurate sCT generation. The multi-sequence fusion approach by Li et al. [55] addressed this by adaptively combining T1 and T2 data using attention mechanisms, improving both intensity and structural accuracy and enabling more precise dose calculations. Similarly, Florkow et al. [56] showed that different multi-channel gradient echo combinations influence sCT quality. Integrated with DL, their approach achieved high accuracy in various tissue regions.

#### 3.3.2. CT Synthesis from CBCT

Image-guided adaptive radiotherapy relies on daily CBCT, but scatter, noise, and inconsistent HU limit its direct use for accurate dose calculation. Recently, latent diffusion models—particularly Denoising Diffusion Probabilistic Models (DDPMs)—have emerged as a promising alternative to GAN-based approaches for CBCT-to-sCT synthesis. Compared with adversarial training in GANs, the iterative denoising objective of diffusion models avoids mode collapse and provides more stable optimization, which in practice translates into better preservation of fine anatomical detail and more faithful modeling of tissue-specific HU distributions. The trade-off is significantly higher inference cost (multiple sampling steps per image); although, recent latent-space and consistency-model variants have begun to close this gap. For example, Zhang et al. introduced a texture-preserving diffusion model that improved the fine-structure consistency [57], while Chen at al. explored localized or region-specific diffusion to enhance lung-tissue density estimation [58]. Furthermore, recent frameworks such as patient- and fraction-specific lung diffusion models (PFS-LDMs) fine-tune a general diffusion backbone using individual patient anatomy to improve image similarity and HU accuracy compared to non-personalized models [59]. Despite these advances, most current CBCT-to-sCT diffusion models are trained and validated on single-institution datasets, leaving domain shift across CBCT scanner vendors, kVp/mAs settings, scatter-correction algorithms, anatomical sites, and patient body-habitus distributions as a major open problem. Multi-institutional federated training and explicit domain-adaptation strategies represent active research directions but have not yet produced widely benchmarked solutions.

#### 3.3.3. CT Synthesis from Truncated Images

In external beam radiotherapy and online adaptive workflows, onboard imaging often suffers from field-of-view (FOV) truncation, limiting anatomical completeness and dose-calculation accuracy. To address this, deep learning frameworks have explicitly targeted both FOV extension and truncation artifact correction. Early models like Compensation Cycle-Consistent GAN (Comp-GAN) simultaneously demonstrated sCT generation and peripheral anatomy recovery in pelvic and head-and-neck MR scans [60]. Building upon these adversarial foundations, later approaches incorporated explicit anatomical guidance to improve boundary fidelity; notably, the Structure-Completion GAN (SC-GAN) integrated external body masks to enforce anatomically consistent and complete geometry in severely truncated MR images [61]. Similar challenges in pediatric CBCT have been tackled with CycleGAN-based frameworks, though full clinical validation is still ongoing [62]. The methodological evolution in this subfield has been dominated by adversarial frameworks (Comp-GAN, SC-GAN, CycleGAN-based approaches), which demonstrated proof of concept for joint synthesis-and-extrapolation but face training-stability and peripheral-fidelity limitations. More recently, diffusion-based outpainting has been explored in adjacent medical-image-synthesis settings and represents a natural extension, although peer-reviewed studies specifically applying diffusion outpainting to radiotherapy CBCT/MR truncation correction remain limited at the time of writing. For both GANs and diffusion models, key open challenges remain: validation on truly out-of-distribution truncation patterns, dosimetric impact characterization, and prospective clinical evaluation.

#### 3.3.4. Missing Modality Completion

In clinical practice, ideal multimodal imaging is often unavailable due to equipment, patient, and time limits, resulting in missing modalities [63]. AI offers a promising solution by enabling the synthesis of absent modalities from available data, thereby preserving diagnostic and treatment quality. MedGAN, proposed by Armanious et al. [64], established a general medical image translation framework (e.g., MRI-to-CT, PET-to-CT) using multi-scale discriminators and perceptual loss to ensure the realism of generated images, enabling a single model to address multiple clinical needs. Yang et al. [65] proposed a unified framework to handle arbitrary missing modality situations and achieved flexible completion through shared encoders and modality-specific decoders. Even with 1–2 missing modalities, segmentation performance was maintained—an important robustness for practical applications.

## 4. AI in Radiation Treatment Planning

### 4.1. Multimodal Image Registration

Image registration is essential in radiation therapy for aligning images across time points and modalities, supporting treatment planning and dose accumulation. By leveraging prior knowledge, deep learning has improved registration speed, robustness and accuracy while shifting the field from traditional iterative optimization to end-to-end learning [66,67,68], especially for complex multimodal data. A central challenge in multimodal image registration is the significant appearance variability between imaging modalities. CT highlights bone but lacks soft tissue contrast, MRI offers superior soft tissue detail and functional information without electron density, and PET provides functional information with low spatial resolution. Establishing reliable correspondences among these differences remains a long-standing challenge in this field.

While DL-based image registration can be implemented through supervised or unsupervised frameworks, unsupervised approaches are often more clinically relevant as they do not require ‘ground-truth’ deformation fields for training—a significant bottleneck, especially in multimodal registration tasks. Unsupervised methods learn deformation fields by directly optimizing a loss function that combines image similarity with regularization to ensure physically plausible and smooth deformations. Duan et al. [69] provided a comprehensive survey of recent advances in unsupervised DL-based medical image registration and demonstrated the outstanding performance across diverse imaging modalities and anatomical regions. Their review highlights technical improvements using direct regression networks, feature-based learning architectures and multi-state refinement strategies, underscoring the growth and versatility of DL methods in certain clinical practices.

Traditional multimodal registration methods rely on handcrafted similarity metrics, such as mutual information, which struggle with complex inter-modal relationships. Fan et al. introduced adversarial learning for registration [70], in which a generator learns to produce registration fields while a discriminator distinguishes between aligned and unaligned images. Instead of designing complex similarity measures, the network learns what “alignment” means. This approach reduced CT-MRI registration error to 2.3 mm but suffers from the instability of adversarial learning. The cross-modal attention mechanism proposed by Song et al. provides a more stable solution [71] by identifying modality-invariant anatomical correspondences. When combined with a contrastive learning-based pretraining, this method demonstrates improved performance for multimodal image registration.

Uncertainty assessment is essential in DL-based image registration before clinical adoption because the problem is ill-posed and normally without ground truth guidance [69,72]. Quantifying confidence helps avoid overreliance in error-sensitive tasks like contour propagation in adaptive radiotherapy, and dose accumulation for outcome analysis.

### 4.2. Automated Segmentation

Automating radiation target and OAR delineation is a primary application of AI in treatment planning, enhancing both workflow efficiency and contouring consistency [73]. DL models outperform atlas-based methods in accuracy and speed [74]. While 3D U-Net architectures remain a widely adopted high-performing baseline, their reliance on convolution operations can limit the capture of long-range spatial dependencies. Consequently, hybrid CNN–Transformer architectures—such as the Swin UNETR [75], which couples a Swin Transformer-based encoder with a CNN-based decoder—have demonstrated superior performance in delineating morphologically complex tumors by more effectively modeling global context [76,77]. The data-intensive nature of these advanced models is addressed by data-efficient strategies, such as self-supervised pretraining, which learns robust anatomical features from large unlabeled datasets. More recently, large-scale foundation models for medical imaging have shown promise for cross-site and cross-modality generalization with minimal fine-tuning [78].

The gross tumor volume (GTV) represents the visible extent of the tumor. DL models can achieve high accuracy in GTV delineation, though performance varies by anatomical site and imaging modality. For brain metastases on high-contrast MRI, DL achieved a mean Dice Similarity Coefficient (DSC) of 0.92 [79], nearing human performance. For NSCLC on CT, an attention U-Net model attained a mean DSC of 0.82 [80], while a large multi-center study for nasopharyngeal carcinoma on MRI reported a mean DSC of 0.83 [81]. Performance can be lower on CT with poor soft-tissue contrast. The Clinical Target Volume (CTV) includes the GTV plus a margin for subclinical microscopic disease based on clinical guidelines. AI is well-suited for this rule-based task to improve consistency and efficiency [82]. For breast cancer, a 3D-CNN increased guideline adherence from 77% to 91%, saving 24 min per case [83]. Similarly, U-Net models have achieved high accuracy (DSC 0.81–0.90) for complex head-and-neck nodal CTVs, with 99% of contours requiring minimal or no edits [84]. Multimodal imaging further enhances target delineation accuracy. PET/MRI fusion using parallel-encoder networks reached a DSC of 0.73 for cervical tumors [85], while fusing T1-post, T2, and FLAIR MRI sequences is standard for delineating distinct brain tumor components.

Automating OAR delineation is mature and widely adopted, performing best for larger, well-defined organs (lung DSC 0.94–1.00; brain and liver DSC > 0.95) with most auto-contours clinically usable without significant edits [86]. Performance drops for small, mobile structures with poor soft-tissue contrast (esophagus DSC 0.55–0.87; bowel DSC ~0.60), often needing correction [87]. Domain shift across institutions is a major challenge for generalizability, necessitating rigorous site-specific validation. The primary benefit of OAR auto-segmentation includes ~35% reduction in planning time [82], enabling online ART with rapid re-contouring (approx. 1–2 min) that would be impractical to perform manually.

Another benefit of AI-driven segmentation is its potential to mitigate inter-observer variability (IOV), a limitation of manual contouring in radiotherapy precision [74]. Studies show that AI-based segmentation significantly reduces IOV. For instance, one study reported AI contours achieved higher consistency (mean DSC 0.78) than human experts (mean DSCs 0.67–0.72) [88]. While not replacing clinicians, AI automates repetitive tasks, allowing practitioners to focus on complex cases and the critical verification of the AI’s output. Robust validation frameworks (e.g., AAPM TG-384) are essential for patient safety and medico-legal considerations [89]. Overall, AI-driven segmentation can standardize language for defining anatomy, accelerate practice and advance evidence-based radiotherapy.

### 4.3. Automation of Treatment Planning

The automation of radiotherapy treatment planning has evolved through several distinct technological phases. This evolution can be broadly categorized into two major paradigms: Knowledge-Based Planning, which leverages structured knowledge from past clinical cases, and DL-Based Planning, which employs complex neural networks to learn end-to-end solutions directly from raw data.

#### 4.3.1. Knowledge-Based Planning (KBP)

KBP was the first major data-driven approach to automate and standardize IMRT and volumetric modulated arc therapy (VMAT) planning [90]. It leverage the collective experience embedded within a large database of previously approved, high-quality plans to guide new planning [90]. Instead of starting from scratch, KBP systems analyze target-OAR geometry in historical cases to predict the best achievable dosimetry for a new patient with similar anatomy [91].

This approach marked a significant departure from earlier rule-based automation attempts, which used hard-coded, “if-then” logic to script planning actions and struggled with anatomical diversity [90]. KBP is inherently adaptive because its predictions are grounded in real patient data. The core principle is to build a machine learning model that can predict achievable, patient-specific dose-volume objectives from given patient’s anatomical information. These predictions provide a highly optimized and informed starting point for the inverse planning engine, dramatically reducing the need for manual trial-and-error iterations [90].

The KBP workflow involves several key technical steps to translate historical planning into actionable guidance for new plans. It starts with a curated library of high-quality plans, extracts features that describe the patient’s unique anatomy, and then learns the correlation between these geometric features and dosimetry from the historical plans. The most common prediction target in KBP is the Dose-Volume Histogram (DVH) for each OAR [90]. The model learns to predict an entire DVH curve, which represents a set of achievable dose-volume constraints for that organ given its proximity to the target. Some KBP systems may also predict specific dose metrics (e.g., the mean dose to the parotid gland) or, in more advanced implementations, a full 3D voxel-level dose distribution, though DVH prediction remains the most prevalent approach [91].

To build this predictive relationship, KBP systems employ a range of traditional ML algorithms. These include multivariate linear regression, support vector regression, random forests, and principal component analysis (PCA), which is used to reduce the dimensionality of the feature space [90]. More recently, DL techniques have also been incorporated into KBP frameworks to create more powerful and nuanced predictive models [92].

The principles of KBP have been successfully translated into commercial products that are now widely used in clinical practice. The most prominent and extensively studied of these is RapidPlan (Varian Medical Systems) [93]. RapidPlan allows clinics to build their own KBP models using their internal database of patient plans or to leverage pre-existing, validated models shared by other institutions [94,95].

In terms of efficiency, KBP dramatically reduces the number of iterative adjustments required to achieve a clinically acceptable plan. Studies have reported substantial reductions in active planning time, with one systematic review finding that KBP routines for stereotactic radiotherapy could be completed in 10–35 min, compared to 60–90 min for manual planning [96,97]. Another study found that KBP reduced the average planning time for lung plans from 40 to 60 min to just 10–15 min [98,99]. This acceleration of the workflow not only improves departmental throughput but also makes more complex planning techniques, such as those for stereotactic treatments, more accessible [91].

KBP has been shown to consistently produce high quality plans, often comparable or even superior to those created by experienced planners [90]. Numerous studies have documented dosimetric gains such as improved OAR sparing without sacrificing target coverage [100,101]. A key benefit of KBP is the reduction in plan variability. The data-driven, semi-automated workflows are less dependent on the individual planner’s experience or the planning time available [102], leading to more consistent and standardized quality of care, making expert “knowledge” available to every patient, and potentially reducing healthcare disparities.

However, the effectiveness of KBP is not without its nuances. The performance of any KBP model is fundamentally constrained by the training library quality. A study that compared two lung cancer KBP models showed that using plans from an automated multi-criteria optimization system produced significantly better results, with lower heart and esophagus doses, than standard manual plans [103]. This finding reveals a deeper implication of KBP technology. While it can democratize expertise by disseminating the planning practices of a given institution, it may not be a universal equalizer across institutions. Centers with a history of superior planning expertise can build superior KBP models, potentially creating a feedback loop that widens the quality gap between leading centers and those with less experience. Therefore, the input data quality is the single most important determinant of a KBP model’s clinical value. When the KBP development process keeps the end user preferences and application in mind, Court et al. has found high clinical acceptability for the Radiation Planning Assistant (RPA), a web-based treatment planning system (TPS) for low-resource clinics [104,105]. In these instances, considering a design goal for internal and external validation (e.g., 90% clinically acceptable) during phased development can be valuable for clinical tools that will eventually be deployed.

#### 4.3.2. Deep Learning-Based Planning

While KBP represented a major leap forward, the latest frontier in automated treatment planning is defined by the application of deep learning. DL-based methods signify a conceptual shift in how AI interacts with the planning process. KBP can be understood as an AI system that guides the planning process by providing intelligent, data-driven objectives for an optimization engine. In contrast, end-to-end DL models aim to generate the final desired output—such as the complete 3D dose distribution or the machine delivery parameters—directly from patient anatomy. This transition from a guidance tool to a generative one represents a fundamental change in the human–AI interaction model and enables a much higher degree of automation.

The most mature application of DL in treatment planning is the 3D voxel-wise dose prediction. This task is framed as an image-to-image translation problem, where the input is a multi-channel 3D image representing patient anatomy (e.g., CT scan and binary masks of the planning target volume (PTV) and OARs), and the output is a voxel-wise dose distribution [106,107,108]. CNNs are exceptionally well-suited for this task due to their ability to learn spatial hierarchies of features from image data. Numerous studies have successfully developed and validated 3D U-Net-based models for dose prediction across various cancer sites, including head-and-neck, breast, lung, and prostate cancer [106,109]. To further improve accuracy of predictions, researchers have incorporated more advanced architectural elements. Residual connections (from ResNet architectures) help to train deeper networks and mitigate the vanishing gradient problem, while GANs refine the predicted dose distributions to make them more realistic and physically plausible [110,111,112].

The validation of these complex 3D models is a critical step. Performance is typically evaluated using a suite of metrics that compare the predicted dose distribution to clinical ground-truth. These include voxel-wise metrics like the MAE, dosimetric metrics derived from the predicted DVH curves (the “DVH score”), and spatial accuracy metrics like the 3D gamma passing rate, which assesses both dose difference and distance-to-agreement [109]. To facilitate standardized, objective comparison between different models, public challenges have been established, with the AAPM Open Knowledge-Based Planning (OpenKBP) Grand Challenge being a landmark initiative that provided a large, multi-institutional dataset for head-and-neck cancer dose prediction [108].

Given the predicted dose distribution, a second DL architecture is required to predict machine parameters for each control point, such as gantry and collimator angles, Multi-Leaf Collimator (MLC) shapes, and monitor units. One strategy is to train a DL model to predict the optimal fluence maps for each treatment beam. The input to such a model could be the desired dose distribution or the patient anatomy from a beam’s-eye-view perspective [112,113,114,115]. Once the fluence maps are predicted, they can be passed to a leaf-sequencing algorithm to generate the final deliverable plan.

A more ambitious goal is the direct prediction of the MLC leaf positions and monitor units (MUs) that define a VMAT arc. Recent work has explored using a combined DL architecture to predict MLC apertures and MUs at each control point for a single VMAT arc [116,117]. Deep Reinforcement Learning is also being investigated as a powerful technique to solve the sequential decision-making problem of determining the optimal MLC sequence throughout a gantry rotation [118]. These end-to-end approaches promise the fastest possible plan generation but are also the most challenging to develop and validate.

#### 4.3.3. Summary of Automatic Treatment Planning

The automation of radiotherapy treatment planning has progressed through two major paradigms: KBP-based and DL-Based Planning. KBP leverages historical high-quality plans to predict achievable dose objectives for new patients, streamlining planning and improving consistency. Commercial systems like Eclipse^TM^ RapidPlan^®^ have successfully implemented KBP, though its effectiveness depends heavily on the quality of the training plans in its library. This dependency highlights KBP’s role in disseminating expertise but also its potential to perpetuate quality gaps between institutions. DL-Based Planning represents a more advanced, end-to-end approach, directly generating treatment plans. Initially focused on predicting 3D dose distribution using models like U-Net, this paradigm treats planning as an image-to-image translation problem. The ultimate ambition of this field is the direct prediction of deliverable machine parameters, such as MLC positions and MUs for multiple VMAT arcs. While these end-to-end models promise the highest degree of automation and efficiency, they also present the most significant challenges in development, validation, and clinical implementation.

### 4.4. Dose Tracking and Accumulation

Accurate assessment of delivered and cumulative dose is essential in ART and reirradiation to evaluate tumor control and normal tissue toxicity. Anatomical changes during treatment render simple dose summation inaccurate. Deformable image registration (DIR) provides the necessary technical foundation to warp and sum dose fractions or treatment courses onto a common anatomical frame. Yet, this process remains a complex challenge, extending beyond image registration to include the selection of mapping methods, uncertainty quantification and other aspects to ensure clinical validity. The introduction of DL tools address DIR challenges but raises questions about physical plausibility. Smolders et al. [119] proposed a DL-based uncertainty prediction framework for DIR that jointly optimized image similarity and quantified uncertainty of dose accumulation for online ART. By developing supervised and unsupervised models to estimate the Gaussian uncertainty of deformable vector fields, this method aided reliable contour propagation and dose mapping, with promising results for head-and-neck and lung OARs. Zhong et al., highlighted deformable dose accumulation as essential for ART [120], while the review by Nenoff et al. [72] provided comprehensive recommendations and practical guidance on DIR uncertainties across various anatomical sites to support clinical deployment.

## 5. AI in Motion Management and Delivery

### 5.1. Motion Management in Image Registration Stage

Patient motion is a critical challenge in high-precision radiotherapy, prompting the development of four-dimensional imaging like 4D-CT, 4D-CBCT, and 4D-MRI to explicitly capture respiratory motion patterns. For example, 4D-CBCT was pioneered to reduce geometric uncertainties in SBRT treatments, enabling frameless motion management [121]. Clinical studies using on-board 4D-CBCT have revealed substantial inter-fraction variation in tumor motion that deviates from planning 4D-CT, necessitating frequent motion assessment and adaptation [122]. Traditional 4D-CBCT, however, relies on phase binning under an assumption of periodic motion, which severely limits the number of projections per phase. This sparseness leads to image quality degradation and undersampling artifacts unless one prolongs the scan or increases dose [123]. Consequently, recent research has focused on AI-driven DIR approaches to improve deformation vector field (DVF) estimation for motion compensation, without sacrificing imaging speed or quality. Huang et al. combined a U-Net CNN with a simultaneous motion estimation and image reconstruction (SMEIR) framework [124] to accurately solve lung DVFs using only 80 projections per phase in 4D-CBCT, overcoming failures of traditional intensity-based methods in low-contrast images [125]. In another study, Zhang et al. demonstrated that preprocessing phase images with a CNN to suppress artifacts (e.g., streaks from undersampling) can improve subsequent deformable registration and motion modeling [126]. They introduced a DL-based groupwise registration framework into the 4D-CBCT reconstruction pipeline to jointly align all respiratory phases at once, rather than pairwise [127], reducing accumulated alignment error and inter-phase inconsistencies. This integrated framework effectively resolved inter-phase motion while maintaining phase-specific detail and achieved near-real-time 4D reconstruction for online adaptive use. Such innovations highlight how neural networks can augment conventional registration, not only speeding up computation, but also improving the fidelity of motion alignment crucial for accurate dose delivery.

**Figure 8:**
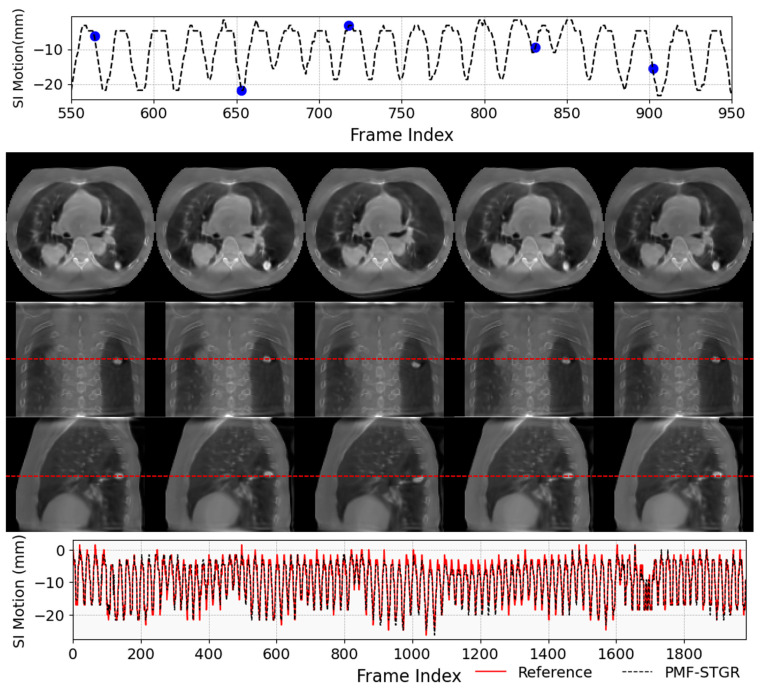
PMF-STGR reconstructed dynamic CBCTs. Row 1: superior-inferior (SI) motion trace; blue markers indicate the motion states visualized. Rows 2–4: reconstructed CBCT volumes at the selected states. Row 5: SI motion trajectory estimated by PMF-STGR (solid) versus a reference trajectory extracted with the Amsterdam–Shroud (AS) projection method (dashed). Figure is reproduced from [Xie, J. et al. [128] Time-resolved dynamic CBCT reconstruction using prior-model-free spatiotemporal Gaussian representation (PMF-STGR), Phys. Med. Biol., 2025] under CC BY 4.0.

Beyond improving iterative registration, recent work formulates motion as continuous DVFs learned jointly with the image, so that respiratory trajectories are recovered directly from treatment-time data using implicit neural models. In STINR, multi-layer perceptrons are employed to encode a reference CBCT and temporal coefficients to parameterize the time-varying DVFs that warp this reference across the breathing cycle, with a PCA motion model derived from the prior 4DCT as guidance. The result is a patient-specific model that captures irregular motion patterns and supports contour propagation, tracking, and dose accumulation. Extended to MRI, STINR-MR couples spatial (reference 3D MR) and temporal INR that encodes the intra-scan DVF, using a joint reconstruction-registration objective. The DVF can be regularized by PCA derived either from prior 4D-MRI or directly from the current cine data, enabling high-temporal-resolution motion fields suitable for gating/tracking and for dose-aware accumulation across time. To reduce reliance on external priors, PMF-STINR learns the motion directly from onboard x-ray projections using a one-shot, prior-model-free scheme. A data-driven B-spline motion model infers motion basis components (MBCs) during optimization, yielding dynamic DVFs/CBCTs without phase sorting/binning and pre-acquired anatomical or motion models, which is advantageous for day-of-treatment motion assessment and adaptive decision-making. Digital phantom and patient studies show robust recovery of complex motion with this approach. A recent prior-model-free spatiotemporal Gaussian representation framework (PMF-STGR) replaces the INR with 3D Gaussian primitives for both the reference CBCT and the learned MBCs, enabling patient-specific dynamic DVFs from onboard projections with ~50% shorter optimization/reconstruction time and an approximately halved GPU memory footprint, while yielding less-blurred images and similar/better motion accuracy than PMF-STINR, thereby improving clinical practicality. Figure 8 shows one example of PMF-STGR reconstructed dynamic CBCTs obtained by deforming reference CBCT via dynamic DVFs [128]. With AI, motion-resolved 4D-CBCT/MRI and dynamic CBCT/MRI now deliver higher-fidelity images together with more accurate, patient-specific motion fields, enabling robust characterization of respiratory trajectories for planning, on-board adaptation, and delivery. The resulting DVFs serve as the backbone of ART, supporting auto-segmentation, organ motion estimation, and dose accumulation [129,130,131], so that coverage and OAR sparing can be evaluated on the anatomy that actually occurred, not a static surrogate. Coupled with AI-powered fast (even real-time) dose estimation [132,133,134,135], these DVFs close the loop from motion measurement to dosimetric decision-making, paving a practical path toward real-time dose adaptation during treatment.

### 5.2. Motion Management During Treatment Delivery

Accurate motion management in radiotherapy before beam-on is crucial to ensure the planned dose is delivered to the tumor with minimal margins. During the pre-treatment phase, various geometric uncertainties can arise due to setup errors, respiratory motion shifts, and anatomical changes. Recent advances in AI and DL have been leveraged to reduce these pre-treatment uncertainties, improve patient positioning, and support adaptive decisions.

#### 5.2.1. Uncertainty in Pre-Treatment Setup and Motion

Even with careful planning, a patient’s anatomy and position at treatment often deviate from the simulation images used for initial planning. Common sources of uncertainty include setup errors, baseline shifts in breathing, and anatomical changes such as weight loss or tumor volume change between planning and treatment. AI-driven image registration is a major tool to mitigate setup uncertainty by aligning on-board images (e.g., daily cone-beam CT or planar X-rays) with the planning CT. CNN-based DIR networks, such as VoxelMorph-style models [136], can learn to predict the spatial transformation that maps daily imaging to the planning anatomy [137]. Lee et al. present Seq2Morph that takes a planning CT and up to six weekly CBCTs and outputs DVFs for all registration pairs in one pass. Trained and cross-validated on 50 lung cancer patients, the network achieved accuracy comparable to the state-of-the-art large deformation diffeomorphic metric mapping algorithm but at clinical speeds of 22 s per-patient inference time. AI-based multi-modality registration approaches have also been explored to align MRI/PET with CT for setup, using methods like supervised CNNs [138,139] and even reinforcement learning [140,141].

For patients with respiratory motion management (e.g., gating or breath-hold), the breathing pattern on the treatment day may differ from planning. AI models have been applied to predict tumor motion ranges or baseline shifts during pre-treatment phase. Lin et al. developed a “super-learner” ensemble model [142] consists of random forest, multi-layer perceptron, LightGBM and XGBoost to predict 3D lung tumor motion amplitude from patient features and a free-breathing CT. Their model achieved mean absolute prediction errors in the order of 1 mm in each direction, accurately anticipating the extent of motion seen on 4DCT. The X360 [143] method was proposed to estimate volumetric liver and tumor motion from a single, arbitrarily angled on-board X-ray projection. The method couples DL feature extraction with sequential rigid and deformable registration, and uses a geometry-aware feature pooling layer that projects a mesh of 3D liver surface nodes into the 2D X-ray feature maps according to the known projector geometry. This physics-informed pooling makes the inference effectively angle-agnostic, allowing standard setup images to recover volumetric boundary motion without specialized acquisition. The resulting DVFs and boundary shifts quantify baseline drift and the expected respiratory envelope prior to delivery, informing last-minute couch corrections and selection of breath-hold versus free-breathing. Building on X360, PCD-Liver [144] introduces diffusion-model priors to regularize the 3D motion estimate, improving motion estimation accuracy and robustness to imaging noise. Together, these approaches can inform adapting the setup or choosing an appropriate motion management strategy before treatment.

#### 5.2.2. AI Enhancements in Surface-Guided Radiotherapy (SGRT)

SGRT uses 3D optical surface scanning to position and monitor patients, offering a non-invasive alternative to skin marks or tattoos. In pre-treatment setup, SGRT projects patterned light onto the patient and uses stereo camera systems to capture the patient’s surface in real time [145]. Although SGRT hardware is not inherently AI-based, researchers are developing AI algorithms on SGRT data to enhance its utility. Douglass et al. show that internal cranial anatomy can be estimated from a patient’s external contour alone: a 2D pix2pix GAN, trained on ~15,000 axial MRI slices with paired external masks and validated on 5000 unseen slices, synthesizes high quality “sMRI” from 3D optical surface scans, enabling detection of internal shifts for brain radiosurgery patients without an extra scan [146]. Zhang et al., used a DIR-enhanced deep network to predict internal tumor displacement from optical surface scans, successfully tracking respiration-induced tumor motion via the surface alone [147]. Combining optical surface imaging to offer complementary information, the Surf-X-Bio [148] framework was proposed for real-time 3D liver tumors from a single X-ray projection localization in SGRT. Compared with its predecessor MeshRegNet-Bio [149] method which relied solely on the X-ray projection, Surf-X-Bio demonstrates robustness to large respiratory motions and to scenarios where parts of the liver boundary fall outside the X-ray FOV, yielding more accurate and reliable in/pre-treatment motion estimation.

Additionally, ML has been used to optimize SGRT workflow. De Kerf et al. developed a ML model to predict the necessary couch translations and rotations from the initial surface scan, prior to any manual adjustment [150]. By learning from past patient setups, the model could output an initial couch position with high precision, serving as a near-optimal starting point. Their results showed couch parameters predicted with such accuracy that in most cases only minor fine-tuning was needed, and any large discrepancy (>1.5 cm) was flagged for manual verification. This kind of AI-driven positioning can reduce iterative adjustments, thereby improving patient comfort and treatment output.

#### 5.2.3. Tumor Shrinkage Assessment and Adaptive Planning

During a radiotherapy course, tumors and patient anatomy can change significantly. Tumors may shrink in volume or change shape in response to the delivered dose, and patients can lose weight or experience shifts in organs-at-risk. Deep learning segmentation models can delineate the gross tumor volume in on-board images rapidly, allowing clinicians to quantify shrinkage without laborious manual contouring. In head-and-neck cancer, TransAnaNet [151], a Swin-Transformer and CNN hybrid that utilizes the planning CT, initial CBCT, RT dose, and GTV masks to predict the fourth week anatomy with high image-level and structure-level agreement, offering a patient-specific prior that can be checked during setup and used to schedule ART when warranted. In lung non-small-cell lung cancer, a CNN-plus-RNN model trained on weekly CBCTs forecasted GTV geometry for weeks 3–6 with a high prediction accuracy [152]. By integrating these predictions into weekly re-optimization, target coverage metrics were preserved while mean lung dose was lowered by ~0.5–2.85 Gy across tested cases, demonstrating actionable pre-treatment benefit. Complementing this, a 230-patient nasopharyngeal cohort using an LSTM-GAN predicted the next week’s CBCT, yielding Dice > 0.94 for targets and >0.90 for OARs, with dosimetric deviations under 1 Gy, enabling adaptation to be scheduled in advance rather than reactively [153].

## 6. AI in Adaptive Radiotherapy

ART is an intrinsically image-driven process where every fraction begins with imaging that must be converted into actionable information for re-optimization and safe delivery. Whereas standard image-guided radiotherapy (IGRT) primarily uses images for setup verification, ART requires images of sufficient fidelity for dose calculation, contour propagation/refinement, and plan quality/safety checks—all within minutes. Consequently, AI-powered image assistance is not a convenience but an enabling layer that compresses time, reduces variability, and raises the ceiling of what can be adapted on-couch [154,155,156].

**Synthetic CT and Virtual Simulation**. A key imaging challenge in ART is the need for accurate electron-density information. MRI-guided systems, while offering excellent soft-tissue contrast, lack direct Hounsfield unit data, whereas CBCT images—though widely available—are often degraded by scatter, shading, and truncation artifacts. AI-based image synthesis addresses both limitations by generating planning-quality sCT from MRI or CBCT, as described in Section 3.3. In MR-guided workflows, MRI-to-sCT conversion enables MR-only simulation and MR-only ART, eliminating the need for CT and reducing geometric uncertainty [157,158,159,160]. For CBCT-based workflows, daily CBCT can be converted into a calculation-ready sCT, supporting same-day adaptive planning on conventional linacs and extending ART access beyond MR-Linacs [161,162]. More recently, AI-driven “virtual simulation” uses diagnostic MRIs to generate treatment-ready planning images, as demonstrated in hippocampal-sparing whole-brain RT, significantly reducing preparation time and resource needs [163].

**AI-Assisted Segmentation for ART.** Daily target/OAR updates are a major pacing item in ART. Clinical deployments of deep-learning auto-segmentation have demonstrated large time savings and improved consistency across sites and institutions [164,165,166,167]. For ART specifically, patient-specific and session-aware models leverage prior contours and prior adaptive sessions to maintain longitudinal consistency and reduce editing effort under time pressure [168,169,170]. Emerging multimodal systems—including models that integrate text (reports) and multi-sequence images informed by large language models—are now improving reproducibility for complex or poorly visualized targets [171,172].

**Biology-Aware Adaptation.** AI extends IGRT beyond simple geometry correction into the realm of early biological response assessment. Radiomics and functional imaging data (e.g., diffusion-weighted MRI [DWI] or PET-derived features) can be analyzed by AI models to flag evolving biology—such as treatment response or progression—so that adaptation can be performed accordingly on a daily basis. In this framework, ART serves as the operational layer that converts imaging-derived evidence into therapeutic change. Near-term clinical implementations focus on incorporating biology-informed signals to trigger adaptation and guide target contour adjustments. AI can further extract quantitative imaging signatures and link them to re-optimization [154,155,156,171,172,173,174,175]. At present, biology-aware adaptation remains largely in the clinical trial and exploratory phase, rather than routine clinical practice, although broader adoption is anticipated in the near future.

**Real-Time Image Assistance for Intra-Fraction ART.** The aspirational end-state is intra-fraction adaptation, where plans are updated continuously during beam-on. Realizing this requires AI models that (i) reconstruct useful images from sparse/low-dose data streams, (ii) rapidly denoise and de-artifact, (iii) estimate motion and geometry in near real-time, and (iv) update dose information fast enough to support decision-making. Deep-learning dose engines and geometry-encoded networks now compute accurate dose in sub-second to second-scale windows and can serve as secondary verification at the same cadence—two prerequisites for safe real-time ART [134,176,177,178].

**Workflow Integration (Image-Centric View).** When viewed through the lens of imaging, a practical ART session can be distilled into a sequential workflow: acquiring the daily image, converting it into calculation-ready data such as a sCT or calibrated volume, updating contours, generating and verifying dose with appropriate quality assurance (QA), and finally delivering treatment. AI streamlines the most time-intensive middle steps—image synthesis (MRI/CBCT-to-sCT) [157,158,159,160,161,162,163], contour propagation and refinement [164,165,166,167,168,169,170], and rapid dose computation and verification [134,176,177,178]. By accelerating these processes, AI ensures adaptive workflows compatible with routine clinical throughput while preserving the standards of quality and safety essential to patient care [154].

**Image-Driven Personalization at Scale.** The next phase of image assistance in ART will focus on integrating multi-sequence MR, PET, and longitudinal daily imaging to guide adaptation based on both anatomy and biology, advancing toward truly response-adapted therapy. At the same time, efforts to standardize virtual simulation pipelines will enable rapid initiation of treatment and simulation-omitted workflows for select clinical scenarios. In parallel, the development of fast dose engines and image-based QA methods will provide real-time decision support that is interoperable across different vendors and platforms. As these capabilities mature, ART will transition from a specialist practice to a broadly deployable standard, reframing precision from a static concept of geometric accuracy to a dynamic, patient-specific model of precision care [155,156].

Table 1 summarizes the AI-powered image assistance in ART nowadays. By turning daily images into sCT suitable for dose, enabling rapid and consistent contour updates, and providing near real-time dose computation and verification, AI converts ART from an aspirational concept into a practical, scalable clinical workflow. The path forward—biology-aware and ultimately real-time—will further elevate images from supportive context to decisive inputs that shape therapy in the moment.

## 7. AI in Machine and Patient-Specific Quality Assurance

Radiation therapy is inherently complex, involving numerous steps and various personnel, hardware, and software to ensure accurate treatment delivery. Each step could benefit from QA to reduce the risk of errors. However, with increasing technological advances, the number and complexity of QA tasks continue to grow, making it more important than ever to prioritize the QA tasks we perform and streamline their execution. AI may be used to identify the areas with the highest risk for failure and improve QA efficiency, either by reducing or removing the need to perform certain QA tasks manually. In this section, we discuss the use of AI in machine QA and patient-specific QA (PSQA).

### 7.1. AI in Machine QA

Machine QA in radiation therapy typically refers to the evaluation of the performance of the linear accelerator and its ancillary components, including the electronic portal imaging device (EPID), onboard imaging, and lasers. A host of dosimetric, mechanical, and image quality QA tests are performed at various intervals, as detailed in recommendations such as those in AAPM TG-142 [180]. Many of these QA tests involve time-intensive data collection, require properly maintained and calibrated tools and equipment, and may be subject to human errors when navigating seldom-used software (custom spreadsheets or commercial software) or hardware (detector choice, equipment operation, etc.).

AI can assist in enhancing these machine QAs by improving efficiency, effectiveness, and accuracy. Studies have shown the use of ML in machine QA to predict machine dosimetry, mechanical properties, and image quality. El Naqa et al. used a support vector data description clustering algorithm with EPID images to automatically QA gantry sag, radiation field shift, and MLC offset [181]. Li and Chan et al. applied artificial neural network (ANN) time-series predictive modeling on daily QA results to predict beam symmetry over time [182]. Using water tank beam data measurements from multiple institutions, Zhao et al. built a multivariate regression model to predict, using only data from the 10 × 10 cm^2^ field, the percentage depth doses and profiles of various other field sizes, potentially enabling more streamlined linac commissioning, annual QA, and QA after major equipment upgrade (such as linac ionization chamber replacement) [183]. Osman et al. used MLC log files to train an ANN model to predict MLC leaf positional deviations during dynamic IMRT treatment delivery, which could be integrated into the TPS’s dose optimization and calculation to improve its accuracy [184]. To improve the ML model’s prediction of MLC positional errors, Chuang et al. added MLC motion parameters, including gravity’s effect on MLC motion based on the gantry and collimator angles [185]. Valdes et al. used support vector machine algorithms on OBI images to automatically identify image artifacts [186]. Furthermore, AI can learn from past QA trends to predict machine issues and complement statistical process control, which enable physicists to anticipate failure modes and take preventative actions.

### 7.2. AI in Patient-Specific QA

The goal of PSQA is to verify that the dose of IMRT/VMAT plans calculated by the TPS reflects the dose delivered by the linac. Measurement-based PSQA prior to treatment can detect unexpected machine delivery errors using a phantom combined with ion chamber, film, EPID, or detector array [187]. This can be time-consuming and labor-intensive, and may lack sensitivity to some clinically meaningful errors [187]. AI-based approaches aim to reduce or eliminate measurement-based irradiation by predicting QA results directly—an approach often referred to as “Virtual IMRT QA”. ML models, including Poisson regression, CNNs, and support vector classifiers, have been successfully applied to predict PSQA pass/fail outcomes using historical treatment plans and their associated PSQA results, and some include plan complexity and machine performance metrics [188].

Valdes et al. first introduced virtual IMRT QA using a Poisson regression model to predict PSQA pass rates based on plan characteristics [189], later validated at an independent institution using a different measurement technique [190]. Subsequently, Interian et al. demonstrated comparable performance using CNNs based on fluence maps without expert-defined features [191]. Tomori et al. further applied a fifteen-layer CNN model incorporating input data including PTV and rectum volumes, overlapping region and MU of each field [192]. Nyflot et al. used CNN to detect simulated MLC errors from EPID images [193].

Generally, treatment plan characteristics are expected to influence the PSQA results. Li et al. found that leaf speed, aperture complexity, small apertures, and MU impact the VMAT dose accuracy, depending on the treatment site [194]. Ono et al. used 28 predictor variables, including plan complexity metrics, with neural networks to successfully predict their ArcCHECK PSQA measurements [195]. Li et al. used 54 complexity metrics with their random forest classification model to detect plans that may fail PSQA with high sensitivity [196]. Wall and Fontenot assessed 241 complexity metrics and plan parameters to determine their relative importance when using various ML models to predict PSQA results [197]. Hirashima et al. used a combination of plan complexity features and dosiomics features with a gradient boosting ML technique to predict PSQA results [198]. Moreau et al. found that, for complex plans (such as breast, pelvis, and head-and-neck), the specificity for predicting PSQA results could be reduced with ML, but could be improved with the use of a deep hybrid learning model [199].

Machine performance metrics can also be used to enhance the ML prediction accuracy. To demonstrate the potential of combining machine performance metrics and treatment plan characteristics in developing ML models for PSQA, Granville et al. trained a linear support vector classifier for PSQA and found that among the most predictive features, half came from plan characteristics and half from machine performance metrics [200]. Taking it one step further, machine performance can be incorporated as part of the TPS dose calculations to give a more realistic reflection of plan delivery. Carlson et al. used ML to predict MLC positional errors and incorporated it into the TPS to correct the dose computation, which significantly improved the PSQA pass rate prediction [201].

Virtual IMRT QA has been repeatedly shown to have acceptable accuracy across different brands of linac, TPS, and QA detectors. AI could enable a significant reduction in PSQA workload, allowing physicists to prioritize higher-value QA activities, as recommended by TG-100 [202]. Cavinato et al. estimated an approximately 35% PSQA workload reduction using a regression model for virtual PSQA of helical tomotherapy plans [203]. Adding process improvement methodologies, Lambri et al. used ML and Lean Six Sigma to complement their PSQA approach, and estimated a 70% workload reduction if they only perform measurement-based PSQA for the predicted failures [204]. They developed a decision support system to monitor the plan complexity and expected PSQA pass rate, so that for the 1.7% plans found at risk of failure, they could re-optimize the plan, to improve overall patient safety. Wall et al. estimated a 69.2% workload reduction and an average savings of 32.5 h per month using their virtual PSQA model [205], while Noblet et al. estimated a time savings of 140 h per year using ML-based decision support to detect suboptimal plans [206]. Nevertheless, these represent projected efficiencies, and the actual time savings achievable may vary depending on factors such as implementation strategy, plan complexity, equipment, workflows, etc.

The use of AI in PSQA can serve three purposes: (i) to serve as a secondary QA mechanism to complement measurement-based PSQA results, (ii) to flag the subset of plans that are likely the fail so that measurement-based PSQA only needs to be performed for this subset, and (iii) to be incorporated as part of treatment planning so that all plans would pass PSQA [207].

There are still concerns about the clinical benefit and limitations of AI-based PSQA. Mainly, there is the underlying assumption that the PSQA gamma criteria is a good surrogate for clinically relevant dose errors, highlighting the limitations of the PSQA process itself. Also, if PSQA is reduced or eliminated, the frequency of other machine QA tests may need to be increased, and additional checks of the transfer of TPS plan parameters to the linac should be performed [207]. Another challenge is that the AI-predicted failing PSQA is hard for a physicist to interpret and determine the failure contributions. Hence, AI-based PSQA as a total replacement for measurement-based PSQA has not been fully implemented.

### 7.3. QA of AI Tools

Before implementation of any AI-based QA tools, it is essential to perform a sanity check and understand their limitations. Some of the challenges of AI-based QA systems stem from data quality (such as incomplete or biased data and inconsistent terminology), model adaptability to evolving practice patterns, and modeling limitations (relatively small number and availability of well-labeled training data) [208]. Furthermore, challenges such as validation of model compatibility across institutions, lack of interpretability of AI models and results, insensitivity to rare failure modes not present in the training data, and performance drift of the model over time need to be addressed, to ensure the reliability and effectiveness of AI systems in radiation oncology. Therefore, conventional QA approaches, developed for non-AI systems, may not be fully applicable. Moreover, all AI applications should align with the evolving regulatory landscape and emerging best-practice frameworks to ensure safe, robust and sustained performance in clinical deployment [209].

For AI-based machine QA, a reference dataset of all the institution’s machines’ QA measurements can be gathered so that the model’s output can be monitored over time using real-world setup deviations or real-world machine faults. For AI-based PSQA, the model’s predictive accuracy can be routinely monitored using plans flagged as potentially failing. Furthermore, a monthly re-delivery of a subset of benchmarked plans with varying complexity should be performed [210].

While the ML output for some other areas, such as auto-contouring, can be easily benchmarked, in the area of QA, the goal of introducing AI is usually to eliminate the performance of the QA task; hence, more transparent tools, such as Bayesian networks and regression models rather than DL, are preferred [211]. The use of AI for machine QA and PSQA is promising but many concerns regarding robustness, trustworthiness and clinical relevance remain to be addressed.

## 8. AI in Outcomes and Predictive Analytics

Advanced imaging and AI have rapidly emerged as transformative tools in predicting tumor response and long-term treatment outcomes. Traditional approaches to assess prognosis rely on tumor staging, pathology, and clinical scores, which, while valuable, often fail to capture the full biological and spatial complexity of tumor behavior and response to radiotherapy. Functional and quantitative imaging complement these clinical and histopathological factors by directly characterizing tumor physiology from medical images. For example, FDG-PET reflects tumor glucose metabolism [212], DWI characterizes cellular density by measuring water diffusion [213], and dynamic contrast-enhanced-MRI quantifies vascularity and perfusion [214]. Building on these advances, AI can integrate advanced imaging techniques with high-dimensional data sources—including radiomics, genomics, pathology, serum biomarkers, and advanced imaging—to build predictive models with enhanced accuracy and personalization. By uncovering nonlinear associations and complex patterns that are not discernible through conventional statistical methods, AI-driven models hold promises for guiding personalized treatment strategies and identifying patients most likely to benefit from specific therapeutic regimens.

### 8.1. Prediction of Tumor Response

AI models have shown excellent performance in predicting overall survival (OS) and tumor control following radiotherapy across cancers. A multi-center study in NSCLC showed that radiomics-augmented XGBoost models achieved a C-index of 0.76 for OS prediction, significantly higher than using clinical parameters alone (C-index 0.52) [215]. Liu F et al. reported that DL-based models leveraging contrast-enhanced ultrasound features improved progression-free survival prediction in hepatocellular carcinoma [216]. Kun Z et al. showed that deep features extracted from pathological images enhanced the prediction of 5-year OS in non-surgical cervical cancer patients [217]. Luo et al. proposed a situational awareness Bayesian network that enabled accurate prediction of personalized ART outcomes in lung cancer patients [218]. Cui et al. developed an ANN with composite architectures for local control prediction in NSCLC, which showed superior performance compared with conventional regression-based models [219]. Building on this work, the same group further advanced their approach by integrating multi-omics information into DL architectures, demonstrating that combining radiomic, genomic, and clinical data provided improved actuarial outcome prediction in NSCLC patients [220]. In radiotherapy, accurate recurrence prediction based on pre-treatment images is critical for guiding personalized surveillance strategies and informing treatment intensification. Tang et al. showed CT-based models integrating GTV/PTV radiomic features achieved AUCs (Areas under the Curve) above 0.93 for recurrence prediction in HNSCC [221]. In soft tissue sarcoma, a multi-institutional study proposed a DL-based radiomic nomogram for recurrence prediction, which integrated radiomic features with clinical factors and outperformed clinical models alone in both internal and external validation cohorts [222]. More broadly, multimodal AI approaches have enhanced prognostic accuracy across a range of tumor types, including head-and-neck cancer [223,224,225], breast cancer [226,227], and prostate cancer [228,229,230].

### 8.2. Tumor Recurrence Detection

Traditional imaging often struggles to distinguish post-treatment changes (e.g., fibrosis, necrosis, inflammation) from tumor recurrence. Radiomics and AI-based approaches address this by quantifying subtle imaging phenotypes beyond visual interpretation to improve diagnostic specificity. For instance, in HNSCC, Zhang et al. combined radiomic features from post-treatment PET/CT with clinical factors and achieved an AUROC (Area under the Receiver Operating Characteristic Curve) of 0.94 for predicting early persistence/recurrence [231]. In glioblastoma, post-treatment imaging interpretation remains particularly challenging due to pseudo-progression. Multiple studies have shown that AI and radiomics-based models improve differentiation accuracy and aid clinical decision-making [232,233,234]. Given that subtle textural changes may precede recurrence, delta-radiomics—which evaluates temporal changes between pre- and post-treatment imaging—offers additional predictive power by dynamically monitoring treatment response. Significant benefits have been shown for HNC [235,236], glioma [237], and rectal cancer [238] for predicting recurrence. Furthermore, Cho et al. reported that DL models incorporating longitudinal images outperformed radiomics for predicting tumor progression in brain metastases [239]. Collectively, these studies underscore that quantitative image biomarkers can capture the subtle biological signatures of relapse versus treatment effect, enabling earlier detection and supporting risk-adapted follow-up pathways.

### 8.3. Treatment Toxicity

Normal tissue toxicity prediction is a critical component of radiotherapy outcome modeling, as treatment-related toxicities significantly impact patient quality of life and may constrain the therapeutic dose that can be safely delivered. Traditional toxicity models in radiotherapy are largely based on DVH parameters and normal tissue complication probability (NTCP) models, which remain clinically validated and widely used due to their interpretability and established role in treatment planning. However, these models assume uniform tissue sensitivity and might fail to account for inter-patient heterogeneity. AI and radiomics-based approaches have emerged as complementary tools by integrating patient-specific imaging phenotypes, clinical data, and even genomics. A study on liver SBRT developed a deep neural network for individualized hepatobiliary toxicity prediction, demonstrating enhanced predictive accuracy compared with standard logistic regression models [240]. Talebi et al. developed a ML–based radiomics model to predict radiotherapy-induced cardiotoxicity in breast cancer patients, enabling early identification of individuals at higher risk for adverse cardiac events [241]. Hassaninejad et al. demonstrated that MRI-based radiomic features combined with clinical variables improved prediction of rectal toxicity (AUC 0.79 vs. 0.65 for NTCP models alone), allowing more accurate patient stratification [242]. Furthermore, substantial progress has been reported in AI-driven approaches for predicting other common complications, including xerostomia [243,244,245], esophagitis [246,247,248], and pneumonitis [249,250,251]. Nevertheless, many of these models remain in the early stages of development, with limited external validation and standardization. Therefore, AI-based approaches should be considered complementary to established NTCP models, with future work needed to ensure robustness, generalizability, and clinical integration.

## 9. Challenges and Limitations

AI has demonstrated significant potential to improve cancer care across every phase of radiation oncology, including earlier cancer detection, workflow automation, enhanced consistency and precision, improved clinical outcomes, and more targeted, personalized treatment strategies. However, despite these advances, several critical challenges present obstacles that hinder widespread, safe, and effective clinical adoption. Many current AI models are not yet ready for routine clinical implementation, and substantial scientific, technical, clinical, and regulatory barriers must still be addressed.

**Data-Related Challenges**. AI models require large, high-quality, and accurately annotated datasets for robust training and validation [252]. In radiotherapy, however, substantial heterogeneity in imaging protocols, contouring practices, treatment planning systems, and institutional workflow can introduce bias and limit generalizability. Ensuring data quality and enforcing standardized formats and acquisition protocols are therefore essential. Additional mitigation strategies include multi-institutional training and external validation, harmonization techniques, and robust quality assurance procedures. Although data sharing across institutions can improve model robustness, it raises significant security and privacy concerns [253]. These risks can be mitigated through data de-identification, secure data governance frameworks, encrypted data transfer, and privacy-preserving approaches. Open, curated datasets may help standardize development, but they are often limited in scale and diversity. Similarly, radiomics has not fully overcome barriers to clinical adoption, particularly challenges related to reproducibility, generalizability, and standardization despite ongoing harmonization efforts [254,255,256]. Mitigation strategies include standardized feature extraction pipelines, transparent reporting guidelines, and prospective multi-center validation to ensure consistent performance across diverse clinical settings.

**Model-Related Challenges.** AI models trained on data from a single institution or specific patient populations often perform poorly on external data due to “domain shift” [257]. This lack of interoperability can perpetuate and even amplify existing healthcare disparities and biases. Mitigation strategies include training on multi-institutional and demographically diverse datasets, leveraging federated learning to enable collaborative model development without direct data sharing, and applying domain adaptation techniques to improve robustness across varying imaging protocols and institutional workflows. Furthermore, many AI models—particularly DL systems—function as “black boxes,” making it difficult for clinicians to understand the rationale behind their recommendations [258]. This lack of explainability remains a major barrier to trust and adoption. Mitigation strategies include integrating interpretable model architectures when feasible, using explainable AI (XAI) tools, conducting uncertainty quantification, and implementing human-in-the-loop (HITL) review processes to ensure clinician oversight. Overall, ensuring model generalizability, interpretability, and reproducibility must be a priority in AI development.

**Clinical Integration Challenges**. For AI to be clinically effective, tools must integrate seamlessly into existing workflows and systems, such as treatment planning software, electronic health records, oncology information systems, and hospital management platforms [253]. Prior to integration, rigorous technical and clinical validation is essential to ensure that models are both safe and effective in real-world clinical settings [210]. Comprehensive training for clinical staff is also necessary to prevent misuse, misinterpretation, and overreliance on AI-generated outputs.

**Regulatory and Ethical Considerations.** The rapid evolution of AI models presents a unique challenge for traditional regulatory approval processes, which are designed for more static medical devices. Critical concerns include algorithmic bias [259], which occurs when AI models exhibit systematic disparities in performance due to unrepresentative training data or flawed model design, and automation bias, where clinicians may over-rely on machine-generated decision-making [259,260]. These risks may exacerbate existing healthcare disparities if unaddressed. Mitigating these risks requires proactive strategies as well as clear guidelines and standards. Using algorithmic bias as an example, mitigation approaches include data-level strategies (ensuring training data are representative and unbiased), model-level strategies (designing algorithms that explicitly prioritize fairness), validation and evaluation (rigorously assessing model performance across subgroups), and deployment and monitoring (ensuring fairness persists in real-world use). Frameworks such as the NIST AI Risk Management Framework (AI RMF) can support systematic auditing and governance, while all efforts should align with evolving legal and ethical standards, such as the FDA’s Predetermined Change Control Plan (PCCP). Guidance statements for AI ethics and good practices have been issued by multiple governing bodies and organizations and continue evolve rapidly alongside technical advances [261].

## 10. Future Perspectives and Conclusions

While the clinical integration of AI tools is still in its early stages, AI is already beginning to transform the field of radiotherapy by significantly enhancing precision, efficiency, and personalization. In the near future, AI is expected to see broader implementation in automating repetitive tasks—such as automated contouring and segmentation, intelligent treatment planning, enhanced image guidance, real-time ART, and streamlined quality assurance—thereby reducing human errors, and standardizing high-quality care.

Looking further ahead, the future of AI in radiotherapy extends beyond mere automation. AI is poised to evolve from a passive tool into an active, intelligent partner in the field of radiation oncology. This partnership will revolutionize early cancer detection through advanced imaging analysis, enable fully automated treatment workflows, and improve risk stratification for personalized intervention. By leveraging continuous streams of clinical and imaging data, future AI models will become generalized, robust, and explainable systems capable of continuous learning and adaptation [262]. This will unlock the potential for truly predictive and preventive oncology, making high-precision, personalized radiotherapy a universal reality and dramatically improving patient outcomes.

As noted in this review, deep learning has automated key components of imaging and radiotherapy workflows; however, these systems can remain largely siloed and passive. They typically do not interpret protocol text, cannot explain their recommendations, and rarely coordinate with other tools or clinicians to actively refine treatment plans. Recently, a promising development of large language models (LLMs), particularly when deployed as coordinated agentic AI systems, offer the potential to orchestrate modular deep learning tools, adjudicate outputs with explainable reasoning, integrate multimodal inputs (e.g., protocol text, segmentations, etc.), and introduce redundancy and quality checks—an approach demonstrated in radiation therapy treatment planning by Ahunbay et al. [263]. While LLMs can be susceptible to fabrication, as shown by Omar et al. [264], agentic AI architectures are increasingly being integrated across leading industries, suggesting a pathway toward more coordinated and transparent AI-assisted radiotherapy workflow [265].

While radiomics has demonstrated considerable promise in enhancing cancer detection and refining radiation outcome analysis, especially in scenarios involving extensive imaging data, recent advances in multi-omics research have further highlighted the significant potential of integrating heterogeneous data modalities. The combination of genomics, transcriptomics, proteomics, metabolomics, and radiomics offers a powerful, system-level framework to comprehensively characterize tumor biology, microenvironment, and treatment response mechanisms, enabling the development of more accurate prognostic and predictive models, and highly personalized and patient-specific radiotherapy strategies [219,220,266,267,268]. Future efforts will need to focus on overcoming technical and analytical challenges—such as data harmonization, model interpretability, and clinical validation—to achieve the goal of biologically guided radiation oncology.

Collaboration is essential for the development of effective AI tools in radiotherapy, requiring coordinated partnerships among researchers, clinicians, policymakers, and industry to integrate diverse expertise, enable robust multi-institutional data sharing, and align technological innovation with clinical needs and regulatory requirements. These multidisciplinary efforts, together with the establishment of rigorous standards and the continued advancement of AI technologies, will play a central role in shaping the future of radiation oncology.

This review focused specifically on AI and radiomics applications within radiation oncology where imaging plays a central role. Given the breadth and rapidly evolving nature of this field, several limitations should be acknowledged. First, the bibliometric analysis relied solely on **PubMed**, potentially omitting relevant studies indexed in other databases. In addition, although we used the most relevant and commonly used keywords (as detailed in Section 2), this strategy may not have captured all pertinent studies. Second, the narrative review in Section 3, Section 4, Section 5, Section 6, Section 7, Section 8, Section 9 and Section 10 prioritized conceptual clarity and representative examples—selected via expert-driven searches and citation tracking—rather than systematic exhaustiveness. Finally, no formal quality appraisal of the included studies was conducted. Despite these limitations, this review provides a comprehensive overview of AI applications in radiation oncology and aims to inform clinicians and researchers about key developments and future directions in the field.

## Figures and Tables

**Table 1:** AI-Powered image assistance in adaptive radiotherapy.

Image Assistance Technique	Adaptive Radiotherapy Application	AI Contribution	Limitations/Confidence
MRI -to-sCT (MR-only ART)	Dose-calculation-ready electron density without CT simulation; consistent MR-MR anatomy across fractions	DL synthesis produces planning-quality sCT; eliminates MR-CT registration uncertainties [157,158,159,160]	Performance depends on anatomical site, scanner, and sequence; residual HU uncertainty may affect dose accuracy in heterogeneous regions; not universally validated across all sites
CBCT-to-sCT (Conventional Linacs)	Daily CBCT becomes a planning image for same-day adaptation	DL synthesis mitigates scatter/shading to enable dose calculation on daily anatomy [161,162]	Residual HU inaccuracies persist; sensitivity to truncation and field-of-view limitations; validation varies by site and imaging quality
Digital Simulation	Simulation-omitted workflows for selected indications (e.g., HS-WBRT)	AI converts diagnostic MRI into synthetic CT in treatment-ready position and extrapolates the missing anatomy for planning; faster time-to-treatment [163,179]	Dependent on registration accuracy and completeness of anatomical representation; sensitive to positioning differences between diagnostic and treatment setups; currently limited to selected indications
AI Auto-Segmentation (ART-aware)	Rapid daily updates of targets/OARs; longitudinal consistency	Clinically deployed DL tools with patient-specific/session-aware refinement [164,165,166,167,168,169,170]	Requires physician oversight and correction; performance varies across structures, image quality, and anatomical changes. A key clinical challenge is the potential discrepancy between model-generated contours and physician- or institution-specific contouring protocols. Careful evaluation and workflow validation are necessary to ensure both clinical accuracy and user acceptability.
Biology-Aware Imaging (Radiomics/Functional)	Early response detection to inform objective setting and adaptation triggers	AI extracts imaging signatures and ties them to re-optimization logic [171,172]	Primarily investigational with limited prospective validation; reproducibility and generalizability across institutions remain active challenges
Fast Dose Engines and Image-Centric QA	Near real-time dose computation and secondary verification to support on-couch decisions	DL dose models and geometry-encoded networks compute/verify dose at sub-second to second scales [134,176,177,178]	Requires validation against conventional dose calculation methods; robustness and interpretability remain ongoing areas of investigation

Abbreviations: ART—adaptive radiotherapy; DL—deep learning.

## Data Availability

All data in this study were provided in the main manuscript.
